# Transcriptomics analysis of salt stress tolerance in the roots of the mangrove *Avicennia officinalis*

**DOI:** 10.1038/s41598-017-10730-2

**Published:** 2017-08-30

**Authors:** Pannaga Krishnamurthy, Bijayalaxmi Mohanty, Edward Wijaya, Dong-Yup Lee, Tit-Meng Lim, Qingsong Lin, Jian Xu, Chiang-Shiong Loh, Prakash P. Kumar

**Affiliations:** 1 Department of Biological Sciences, National University of Singapore, 14 Science Drive 4, Singapore, 117543 Singapore; 2 NUS Environmental Research Institute (NERI), National University of Singapore, #02-01, T-Lab Building, 5A Engineering Drive 1, Singapore, 117411 Singapore; 3 Department of Chemical and Bimolecular Engineering, National University of Singapore, 4 Engineering Drive 4, Singapore, 117585 Singapore; 4 Department of Genome Informatics, Research Institute for Microbial Diseases, Osaka University, Osaka, 565-0781 Japan; 5 Bioprocessing Technology Institute, Agency for Science, Technology and Research (A*STAR), 20 Biopolis Way, #06-01, Centros, Singapore, 138668 Singapore; 6 Centre for BioImaging Sciences, National University of Singapore, 14 Science Drive 4, Singapore, 117557 Singapore

## Abstract

Salinity affects growth and development of plants, but mangroves exhibit exceptional salt tolerance. With direct exposure to salinity, mangrove roots possess specific adaptations to tolerate salt stress. Therefore, studying the early effects of salt on mangrove roots can help us better understand the tolerance mechanisms. Using two-month-old greenhouse-grown seedlings of the mangrove tree *Avicennia officinalis* subjected to NaCl treatment, we profiled gene expression changes in the roots by RNA-sequencing. Of the 6547 genes that were differentially regulated in response to salt treatment, 1404 and 5213 genes were significantly up- and down-regulated, respectively. By comparative genomics, 93 key salt tolerance-related genes were identified of which 47 were up-regulated. Upon placing all the differentially expressed genes (DEG) in known signaling pathways, it was evident that most of the DEGs involved in ethylene and auxin signaling were up-regulated while those involved in ABA signaling were down-regulated. These results imply that ABA-independent signaling pathways also play a major role in salt tolerance of *A*. *officinalis*. Further, ethylene response factors (ERFs) were abundantly expressed upon salt treatment and the *Arabidopsis* mutant *aterf115*, a homolog of *AoERF*114 is characterized. Overall, our results would help in understanding the possible molecular mechanism underlying salt tolerance in plants.

## Introduction

Salinity is a major environmental stress impeding plant growth and productivity^1, 2^, thus affecting about 20% of the cultivable and about 50% of the irrigated lands worldwide^3^. It imposes two kinds of stresses to plants; osmotic stress arising from the reduced water availability due to increased osmotic pressure, and ionic stress due to the increase in the levels of toxic ions like Na^+^ and Cl^−^ leading to ionic imbalance^4^. In this regard, mangrove plants are an important class of halophytes that grow in high saline environment. Several mangrove trees have been shown to reach an optimal growth at salinities of 5–25% of standard seawater^5^. To survive under such saline condition arising from the fluctuating seawater levels, the mangrove plants have developed various morphological and physiological adaptations such as salt secretion via salt glands on the leaves, compartmentalization of salts, accumulation of osmolytes, and salt exclusion (ultrafiltration) by roots^4, 5^. Despite all these ecologically important characteristics, the molecular mechanisms that enable them to adapt and grow in the harsh intertidal habitats remain unknown partly due to the lack of genome sequencing and genomic resources.

In general, salt tolerance is brought about by the interplay of multiple genes, which involves many physiological, biochemical, and molecular processes^1, 6^. Over the past decade, efforts have been made to understand this complex mechanism by profiling the global gene expression patterns in various plant species. In the beginning, most of the molecular insights were obtained using the glycophytic model plant *Arabidopsis* ^7, 8^. Additional work with important crop plants such as rice^9, 10^ and maize^11, 12^ led to the identification and characterization of a number of salt-responsive genes. Such studies also unraveled various signaling pathways and the importance of regulation of expression of specific genes associated with salt tolerance^13, 14^. Important signaling pathways identified included the salt overly sensitive (SOS) pathway, phytohormone signaling pathways (ABA-, auxin- and ethylene-mediated) and Ca^2+^-signaling pathways, which helped in understanding the molecular aspects of salt tolerance^15–17^. Later, transcriptome analysis was also performed on many non-model plants such as cotton, *Populus*, chickpea and coconut^18–21^. Despite the vast molecular data available on glycophytes, the major limiting factor is their inability to survive under high salinity, and therefore, such studies are not sufficient to understand the key genes/pathways associated with salt tolerance. Whereas, halophytes such as mangroves growing in high salt environment serve as ideal candidates for exploring the molecular mechanisms underlying salt tolerance. Hence, researchers have tried to focus on analyzing and understanding the transcript profiles of several halophytes such as *Medicago*, *Mesembryanthemum*, *Thellungiella*, *Aeluropus*, *Atriplex*, *Salicornia* and *Suaeda* leading to identification of salt-responsive genes such as those encoding antiporters (NHX, SOS, HKT, VATPase), ion channels (Cl^−^, Ca^2+^, aquaporins), and antioxidant enzymes (APX, CAT, GST, BADH, SOD), many of which have been used for developing salt tolerant crops^4, 22–27^. Nevertheless, to date, limited data on mangrove transcriptome and microarray analysis have been available, which mainly focus on the salt excluders such as *Bruguiera*, *Rhizophora* and *Heritiera* ^28–32^. Transcriptomic studies on salt secretor mangroves are scarce and only two such studies have been attempted so far involving leaf of *Avicennia marina* and root of *Sonneratia alba* ^33, 34^. A mangrove transcriptome database is currently available for species such as *Avicennia* (*A*. *alba*, *A*. *marina*, *A*. *bicolor*, and *A*. *schaueriana*), *Bruguiera*, *Rhizophora* and *Ceriops* ^32^. However, such information for *A*. *officinalis* is not available.

*A*. *officinalis* is an ecologically important fast-growing mangrove tree species with a widespread distribution throughout Asia and especially Southeast Asia^35^. This species has a remarkably high degree of salinity tolerance with unique characteristics such as salt secreting salt glands on the leaves^36^ and efficient salt filtration at the roots (~95%) by means of enhanced hydrophobic barrier deposition, which prevents non-selective apoplastic ion uptake^37–39^. Besides, they use organic solutes to adjust cellular osmotic potential and demonstrate an increase in production of antioxidant enzymes for scavenging reactive oxygen species under high salinity^40^. Although, proteomic^41^ and subtractive hybridization studies^42^ using the leaves of *A*. *officinalis* have identified several proteins and genes related to salt tolerance, such studies have not been attempted so far in the roots.

The availability of novel high-throughput sequencing methods offers a great opportunity to rapidly generate large-scale sequencing data from non-model organisms for transcriptome organization, expression studies (RNA-Seq), molecular marker identification, gene discovery as well as various functional studies^43–45^. In the current study, we carried out a comprehensive transcriptome analysis of *A*. *officinalis* seedling roots based on the Illumina HiSeq™ 2000 platform to provide a valuable molecular data for further understanding of physiological, biochemical and molecular mechanisms of salt tolerance in halophytes. The findings can serve as valuable baseline information to generate new targets for breeding crop plants with enhanced salt tolerance.

## Results

### Illumina sequencing, *de novo* assembly and functional annotation of unigenes

**Figure 1:**
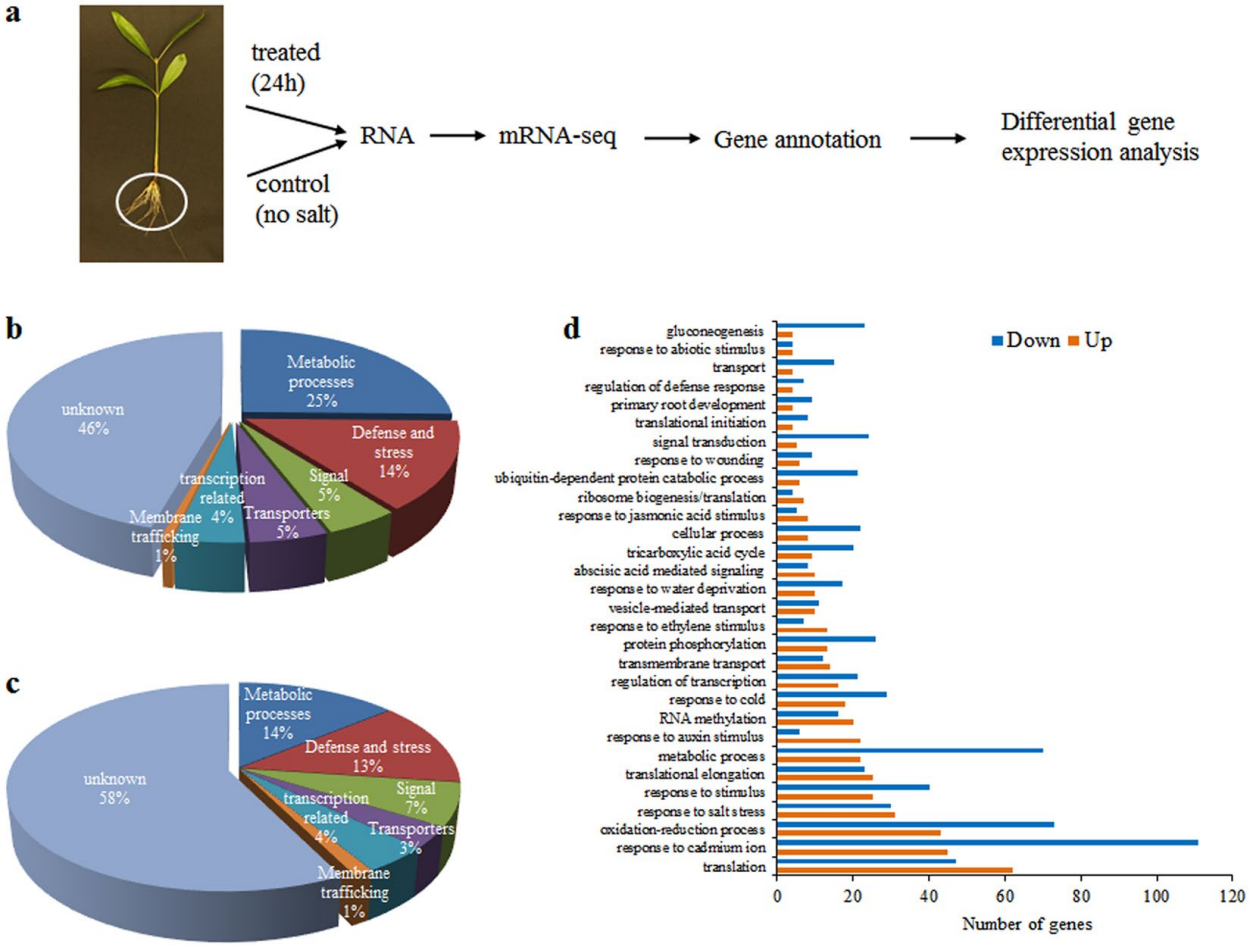
Transcriptome analysis from *A*. *officinalis* roots. (**a**) Schematic of transcriptome analysis from *A*. *officinalis* roots (**b**) percentage of up-regulated and (**c**) down-regulated unigenes classified based on their GO function. (**d**) GO enrichment analysis of DEGs. The top 30 abundantly enriched GO terms that were differentially expressed are represented in the plot. X-axis indicates the number of DEGs enriched. Y-axis indicates the GO term.

Two mRNA-sequencing libraries were generated from control and salt-treated root samples for the comparative transcriptomic analysis in *A*. *officinalis* (Fig. 1a). The sequencing and assembly results are summarized in Table 1. In total, for the control samples 64.31 and 48.96 million clean reads (replicates 1 and 2) and for the salt-treated 62.66 and 48.94 million clean reads (replicates 1 and 2) were obtained. Close to 98% of the clean reads had quality scores higher than the Q20 level (an error probability of 1%) in both samples (Table 1). These high quality clean reads were assembled into contigs (158,671, 134,122 from control replicates 1 and 2; and 143,517, 132,908 from treated replicates 1 and 2), with an average contig length above 350 bp (Fig. S1a and b). After removing the redundancy and aligning the contigs, a comprehensive transcriptome sequence comprising 101,446 all-unigenes was obtained. The average length of unigenes in control and treated samples was 739 bp and 796 bp, respectively, with a N50 of 1238 bp and 1336 bp, respectively (Fig. S1c and d). When the 101,446 all-unigenes were first blasted against Nr database, 71,253 (70%) returned at least one match at the E-value < 10^−5^. *Vitis vinifera* sequences accounted for about 44% of the all-unigenes annotation in our transcriptome study, while 13%, 12% and 7% were retrieved from *Ricinus communis*, *Populus trichocarpa* and *Glycine max*, respectively (Fig. S1e). Remaining 30% of the unigenes did not match to known genes in the database due to the lack of genome and EST information for *A*. *officinalis*. Based on sequence similarity, 52,746 all-unigenes were categorized into 55 GO terms (Fig. S2a). The GO terms were classified into 3 main classes, i.e., biological processes, cellular component and molecular function. Unigene sequences were further subjected to COG classification in order to validate the effectiveness of our annotation process. Out of 71,253 unigenes, 27,436 showed COG classification (Fig. S2b). Among the 25 COG categories, ‘general function prediction only’ was the largest group followed by ‘transcription’, ‘replication recombination and repair’, ‘signal transduction mechanisms’ and ‘post translational modification, protein turnover, chaperones’. The three smallest groups were ‘cell motility’, ‘extracellular structures’ and ‘nuclear structure’. Finally, KEGG pathway analysis was performed to assign biological pathways to all-unigenes. In total, 42,662 unigenes were assigned to 128 KEGG pathways. The major KEGG categories belonged to metabolic pathways, biosynthesis of secondary metabolites, plant-pathogen interaction, plant hormone signal transduction and spliceosome (Table S1).

**Table 1:** Overview of the transcriptome analysis of *A*. *officinalis* roots: Summary of transcriptome sequencing and assembly results of two replicates are presented in the table.

	Control		Salt-treated	
	1	2	1	2
Total clean reads	64,315,388	48,961,920	62,661,642	48,945,928
Total clean nucleotides	5,788,384,920	4,406,572,800	5,639,547,780	4,405,133,520
Q20 percentage	97.98%	98.96%	97.99%	98.93%
GC percentage	46.86%	46.89%	46.66%	46.96%
Total number of contigs	158,671	134,122	143,517	132,908
Mean length of contigs (bp)	360	399	374	393
Total number of unigenes (bp)	112,094	107,138	104,707	105,308
Mean length of unigenes (bp)	739	842	796	757
N50 of unigenes	1238	1410	1336	1263
Distinct clusters	43,099	45,995	43,422	42,358
Distinct singletons	68,995	61,143	61,285	62,950

N50: 50% of the assembled bases were incorporated into sequences with length of N50 or longer.

### Identification and functional classification of DEGs

The analysis showed that 1404 unigenes were up-regulated and 5213 unigenes were down-regulated, while a large portion of the unigenes were not differentially expressed upon salt treatment in *A*. *officinalis* roots. To better understand the relevance of gene expression profile, the DEGs were grouped into six major classes based on their biological functions. About 45% of up- and 60% of down-regulated genes could not be classified based on their functions and hence were labeled as unknown. As shown in Fig. 1b and c, the major classes of genes identified were predicted to be involved in metabolic processes (up 25%, down 14%), defense and stress response (up 14%, down 13%), signal transduction (up 5%, down 7%), transport (up 5%, down 3%), transcription-related processes (4% in both) and membrane trafficking (1% in both). Among the metabolic processes class, genes involved in metabolism of glycerophospholipid, starch and sucrose, glycolysis, ether lipid, TCA cycle, oxidative phosphorylation and pyruvate were significantly regulated by salt treatment. Under defense and stress response, various genes encoding peroxidases, chaperones, cytochrome P450s, heat shock proteins, disease resistance proteins and ubiquitin-conjugated proteases were either up- or down-regulated. Catalases and glyoxylases were only up-regulated and NADH dehydrogenases, hydroxylases, reductases, superoxide dismutase and redoxins were found to be down-regulated. Within signal transduction class, genes encoding calmodulins (CAMs), calcineurin B-like proteins (CBLs), CBL-interacting serine/threonine-protein kinases (CIPKs), LRR family proteins, mitogen activated protein kinases (MAPKs), proline-rich receptor-like protein kinases (PERKs) and serine/threonine-protein kinases were found to be differentially regulated. Genes for rac-like GTP binding proteins, ras-related proteins, serine/threonine-protein phosphatases (PP2As) and two-component response regulators were all down-regulated.

About 71 genes related to various transport processes were up-regulated while 170 genes were down-regulated. Differentially expressed transporter genes are listed in Supplemental Table S2. The major classes of up-regulated transporters were ion-, sugar-and osmolyte-transporters and carriers/permeases while, ATPases and ATP-binding cassette (ABC) transporters were down-regulated. The important up-regulated genes encoding ion transporters belonged to the following families: sodium/hydrogen exchangers (*NHX2 & NHX6*), K^+^ transporters (*SKOR & POT13*), cation/calcium exchanger (*CCX3*), ABC transporters, auxin efflux carrier (*PIN6*) and aquaporin (*PIP2-5*). Similarly, some of the down-regulated transporters included K^+^ channels and transporters (*HKT1* and *HAK23*), vacuolar cation/proton exchangers (*CAX*s), plasma membrane (11) and vacuolar (17) ATPases and ABC transporters. Transcription-related processes group included transcription factors (TFs) as well as genes involved in transcription-related processes. Interestingly, ethylene response factors (*ERF*s), auxin response factors (*ARF*s), No Apical Meristem domain-containing factors (*NAC2*), *WRKY*s and basic helix-loop-helix (*bHLH*) TFs were up-regulated in large numbers, while TFs such as *myeloblastosis* (*MYB*), *zinc finger CCCH domain-containing factors*, *GATA*, *bHLH* and *bZIP*s were prominently down-regulated (Table 2). Other differentially regulated TFs include *general transcription factor group*-*E* (*GTE*s), *Trihelix* TF, *TGA1*, *heat stress* TFs and *MADS-box* TF. A small fraction (1%) of genes related to membrane trafficking were differentially expressed. Transcripts of dynamins, snakins, vacuolar protein sorting, vesicle-associated membrane proteins and CSN4 were differentially regulated, while clathrins, syntaxin and t-SNAREs were down-regulated.

**Table 2:** DEGs related to transcription factors in the root transcriptome of *A*. *officinalis*: Transcription factors that were abundantly regulated by salt treatment are presented in the table.

Gene ID	RPKM		log 2 fold change	*p* value	Homologous species
	control	treated			
**ERF**					
Ug35061	1.85	10.79	+2.48	2.77E-56	*V*. *vinifera*
Ug152478	1.79	9.75	+2.35	1.33E-10	*P*. *trichocarpa*
Ug49200	0.25	1.68	+2.51	3.97E-06	*G*. *max*
Ug42955	0.001	0.06	+5.93	0.04	*B*. *distachyon*
Ug65696	1.22	2.71	+1.11	0.04	*V*. *vinifera*
Ug102419	169.34	12.88	−3.69	0.00	*C*. *roseus*
Ug20505	73.65	17.74	−2.02	7.99E-233	*V*. *vinifera*
**ARF**					
Ug119295	0.001	0.05	+5.78	0.04	*V*. *vinifera*
Ug60441	0.001	0.08	+6.45	0.01	*V*. *vinifera*
Ug26596	0.001	0.88	+9.78	0.04	*P*. *trichocarpa*
Ug27026	23.66	0.93	−5.01	6.33E-199	*V*. *vinifera*
**NAC**					
Ug42722	0.06	1.59	+4.69	3.47E-13	*M*. *domestica*
Ug64865	2.18	9.28	+2.00	1.01E-07	*P*. *trichocarpa*
Ug152309	0.00	4.86	+5.96	9.16E-09	*S*. *bicolor*
Ug149916	0.15	4.34	+4.86	4.35E-08	*C*. *variabilis*
Ug83973	1.35	0.09	−3.59	3.45E-11	*P*. *tomentosa*
**WRKY**					
Ug36701	2.03	8.11	+2.07	2.87E-09	*R*. *communis*
Ug100648	0.46	2.12	+2.16	4.02E-08	*V*. *vinifera*
Ug8314	1.80	9.20	+2.41	4.48E-21	*C*. *sativus*
Ug97853	0.001	0.05	+5.59	0.01	*R*. *communis*
Ug132510	2.88	0.11	−4.71	1.46E-07	*B*. *distachyon*
Ug137386	1.50	0.00	−4.86	8.14E-05	*P*. *tomentosa*
**bHLH**					
Ug120810	0.02	0.14	+2.48	0.04	*V*. *vinifera*
Ug20461	0.001	0.08	+6.29	0.04	*C*. *annuum*
Ug144793	0.05	0.34	+2.66	0.03	*V*. *vinifera*
Ug39192	6.77	1.05	−2.56	2.20E-08	*V*. *vinifera*
Ug138470	6.36	0.54	−3.57	8.68E-16	*S*. *lycopersicum*
Ug138814	3.80	0.35	−3.47	9.21E-06	*G*. *max*
Ug71489	1.24	0.25	−2.27	4.50E-05	*V*. *vinifera*
Ug49859	49.25	5.28	−3.16	1.22E-189	*S*. *lycopersicum*
Ug102959	3.01	0.51	−2.76	1.09E-05	*P*. *trichocarpa*
**MYB**					
Ug86066	12.84	2.27	−2.49	1.31E-48	*V*. *vinifera*
Ug3535	4.40	0.91	−2.08	3.04E-05	*S*. *miltiorrhiza*
Ug155113	1.48	0.00	−5.86	1.60E-08	*S*. *tuberosum*
Ug121384	3.93	0.84	−2.07	1.21E-15	*V*. *vinifera*
Ug37122	3.49	0.43	−2.72	1.53E-06	*G*. *max*
**Zinc finger** (**CCCH**)					
Ug112028	0.03	0.46	+3.96	0.00	*V*. *vinifera*
Ug66562	0.26	1.20	+2.18	1.25E-05	*V*. *vinifera*
Ug131052	21.58	1.52	−3.83	1.27E-98	*C*. *reinhardtii*
Ug137919	1.62	0.00	−5.04	2.48E-05	*V*. *vinifera*
Ug135055	6.85	0.53	−3.69	7.80E-44	*O*. *tauri*
Ug136219	2.48	0.00	−4.86	8.14E-05	*S*. *moellendorffii*
**GATA**					
Ug154250	0.25	1.26	+2.16	0.00	*V*. *vinifera*
Ug137615	6.53	1.22	−2.42	9.86E-15	*V*. *vinifera*
Ug10919	11.78	2.22	−2.41	3.81E-23	*N*. *tabacum*
Ug138999	12.13	1.80	−2.69	1.93E-14	*P*. *trichocarpa*
**GTE**					
Ug149801	1.43	7.31	+2.35	1.33E-10	*P*. *sojae*
Ug91768	0.001	0.03	+5.04	0.01	*G*. *max*
Ug134172	3.89	0.21	−4.22	5.51E-14	*A*. *anophagefferens*
**bZip**					
Ug93351	0.00	0.26	+4.85	0.00	*V*. *vinifera*
Ug59417	16.27	3.84	−2.06	1.10E-48	*V*. *vinifera*
Ug131032	5.30	0.10	−5.77	5.48E-28	*G*. *max*
**Trihelix**					
Ug113286	15.46	64.55	+2.03	7.33E-101	*R*. *communis*
**TGA**					
Ug104907	0.14	0.81	+2.86	5.23E-06	*V*. *vinifera*
**Heat stress**					
Ug132833	6.59	0.16	−5.38	1.37E-21	*O*. *sativa*
Ug133562	5.30	0.00	−7.06	5.03E-17	*B*. *distachyon*
**MADS-box**					
Ug134705	5.11	0.39	−3.72	8.31E-18	*P*. *infestans*

Column 1 shows the gene ID, while column 2 and 3 represent the RPKM values for control and treated samples, respectively. Log 2 fold change in expression levels are shown in column 4 while *p* value is given in the column 5. Column 6 indicates the species to which the assembled sequence was blasted in Nr BLAST.

GO enrichment analysis was carried out to further clarify the biological functions of identified DEGs that were enriched in 56 GO terms. Significantly enriched terms under biological processes are; translation, response to cadmium ion, oxidation-reduction processes, response to salt stress, response to stimulus and metabolic process (Fig. 1d). In total, 2628 DEGs were enriched in 122 KEGG pathways, which include 42 metabolic pathways (q-value ≤ 0.05) (Supplemental Table S1). Abundantly enriched biosynthetic pathways include biosynthesis of secondary metabolites (467 genes), phenylpropanoids (53 genes), unsaturated fatty acids (25 genes), valine, leucine, isoleucine (22 genes) and flavonoids (21 genes).

### Experimental validation of DEGs

To assess the reliability of our RNA-sequencing based approach to identify salt-responsive genes in *A*. *officinalis* roots, we monitored expression of DEGs by quantitative real time PCR (qRT-PCR) analysis. From the 75 DEGs tested, about 68 DEGs (~90%) showed general agreement with their differential expression determined by RNA-seq (Fig. S3a and b), suggesting the reliability of the transcriptome profiling data. However, qRT-PCR analysis showed much higher fold change in the expression levels of some of the DEGs compared to the RNA-seq results, while a few (~10%) showed completely contradictory results (Fig. S3c).

### Identification of key salt tolerance-related genes

To better understand the relevance of the transcriptome data obtained from *A*. *officinalis* roots, the key salt tolerance-related genes were identified by aligning the DEG sequences of *A*. *officinalis* roots with published (GEO database) root transcriptome/microarray sequences of *Bruguiera gymnorhiza*, rice and *Arabidopsis* obtained upon salt treatment. While 75 genes were obtained by alignment with rice, 21 and 14 genes were identified by alignment with *Bruguiera gymnorhiza* and *Arabidopsis*, respectively (Table 3). A total of 93 salt tolerance-related genes were obtained after removal of the repetitive genes and these are listed in Table 4. Based on their GO function, these identified genes were predicted to be involved in metabolic processes, defense and stress, signaling, transport, transcription-related processes, trafficking and cytoskeleton. Among the 93 identified genes, 13 were present in more than one dataset (highlighted in Table 4) which indicates that these could play an important role in rendering salt tolerance to plants. However, the importance of other genes cannot be ignored. The roles of some of these identified genes such as, *hexokinase* ^46^, *cationic peroxidase* ^47^, *Trihelix* TF^48, 49^, *NAC domain containing protein* ^50^, *14-3-3* ^51^ and *calmodulin* ^52^ are well studied under salt stress. However, no studies have been carried out on many of the other genes identified. Therefore, further experimental validation would be required to understand the precise roles of these identified genes under salt stress.

**Table 3:** Number of key salt-responsive genes identified from the root transcriptome of *A*. *officinalis*: The sequences of DEGs were aligned with the published (GEO database) transcriptome and microarray data obtained from the roots of *Bruguiera gymnorhiza*, *Arabidopsis* and rice in response to salt treatment.

Species	Hits	Reference	GEO ID
*Bruguiera gymnorhiza*	21	Yamanaka *et al*.^31^	GSE10942
*Arabidopsis*	09	Dinneny *et al*.^104^	GDS3216
*Arabidopsis*	05	Geng *et al*.^105^	GSE46208
Rice	70	Mizuno *et al*.^107^	GSE20746
Rice	05	Cotsaftis *et al*.^106^	GSE14403

**Table 4:** Key salt tolerance-related genes identified from the root transcriptome of *A*. *officinalis*: The DEG sequences of *A*. *officinalis* were aligned with 4 of the published root transcriptome and microarray data that were obtained from the roots of *Bruguiera gymnorhiza*, rice and *Arabidopsis* in response to salt treatment.

Unigene ID	Ref ID	% similarity	e-value	bit score	Gene name
***Metabolic process*** (*Up*)					
**Ug117296**	LOC_Os01g48960.1	76.29	0	693	***Glutamate synthase 1 [NADH]***
Ug150578	LOC_Os07g41750.1	76.69	3.00E-65	250	*40S ribosomal protein S3-2*
Ug150734	LOC_Os03g29460.1	81.61	7.00E-34	145	*60S ribosomal protein L27a-3*
**Ug152117**	LOC_Os01g53930.2	84.16	6.00E-20	99	***Hexokinase-1***
**Ug19670**	LOC_Os11g21990.1	78.11	0	784	***Probable eukaryotic translation initiation factor 5-2***
Ug38087	LOC_Os05g11710.1	75.95	8.00E-45	182	*60S ribosomal protein L11-2*
Ug56565	Bg04-15_E08	77.56	6.00E-78	291	*U1 small nuclear ribonucleoprotein*
Ug62294	LOC_Os11g06750.1	73.26	1.00E-69	265	*60S ribosomal protein L3*
Ug7542	Bg04-20_K13	80.24	8.00E-160	562	*Dihydroxy-acid dehydratase-like*
Ug88245	LOC_Os01g04730.1	73.03	6.00E-15	82	*60S ribosomal protein L26-2*
Ug88349	LOC_Os03g08020.1	80.62	6.00E-133	475	*Elongation factor 1-alpha*
Ug90287	LOC_Os07g07719.1	74.64	2.00E-44	180	*40S ribosomal protein S18*
Ug115561	LOC_Os11g42550.1	94.03	2.00E-21	102	*Probable beta-D-xylosidase 5-like*
(*Down*)					
Ug112641	LOC_Os09g07450.1	86.32	3.00E-21	104	*Flavonol synthase*
Ug121433	LOC_Os01g53900.1	79.13	0	1725	*Elongation factor*
Ug127854	LOC_Os03g08020.1	84.54	9.00E-49	193	*Elongation factor 1-alpha*
Ug128815	Bg05-08_B15	77.08	3.00E-41	165	*Xyloglucan endotransglucosylase*
Ug135361	LOC_Os03g36930.1	71.25	2.00E-26	121	*Eukaryotic initiation factor 4A-3*
Ug138854	LOC_Os03g15780.5	73.51	9.00E-70	265	*Anthranilate synthase component I*
Ug155336	LOC_Os01g46610.1	77.70	9.00E-63	241	*Isocitrate dehydrogenase [NADP]*
Ug15973	AT4G26270.1	77.32	2.00E-100	364	*6-phosphofructokinase 6*
Ug26981	LOC_Os11g47980.1	76.02	5.00E-40	163	*Probable phosphoribosyl formylglycinamidine synthase*
Ug48447	Bg05-18_B13	99.05	8.00E-48	189	*Hypothetical protein*
Ug86979	LOC_Os07g37240.1	81.50	8.00E-167	588	*Chlorophyll a-b binding protein*
***Defense and stress*** (*Up*)					
Ug103082	LOC_Os05g35400.1	77.07	3.00E-15	84	*Heat shock 70 kDa protein*
Ug112102	LOC_Os03g61960.2	73.26	2.00E-16	87	*Ferredoxin-3*, *chloroplastic*
Ug124338	LOC_Os01g72260.1	85.94	5.00E-10	67	*Cytochrome P450 94A1*
Ug128790	LOC_Os09g39500.1	86.38	4.00E-60	231	*Ubiquitin-60S ribosomal protein*
Ug150095	LOC_Os05g38530.1	81.65	3.00E-45	182	*Heat shock 70 kDa protein*
Ug150096	LOC_Os11g47760.5	78.49	5.00E-44	178	*Heat shock 70 kDa protein*
Ug150526	LOC_Os11g26850.3	79.02	3.00E-34	147	*Adenosyl homocysteinase*
Ug153937	LOC_Os08g43640.3	78.22	8.00E-150	531	*Probable 26 S proteasome non-ATPase regulatory subunit 3*
Ug24565	LOC_Os03g16860.2	84.85	4.00E-40	165	*Heat shock 70 kDa protein*
**Ug2927**	Bg04-11_J19	73.80	6.00E-62	237	***Monosaccharide-sensing protein 2***
Ug71715	AT4G31990.4	79.25	0	830	*Aspartate aminotransferase*
**Ug74595**	LOC_Os02g14430.1	74.71	7.00E-12	73	***Cationic peroxidase 1***
Ug77739	LOC_Os06g05240.1	77.27	5.00E-34	147	*Carboxypeptidase D-like*
Ug83277	Bg01-04_K24	80.69	5.00E-51	200	*Uroporphyrinogen decarboxylase*
**Ug9510**	LOC_Os03g16030.1	83.22	9.00E-74	278	*17*.*3 kDa class I heat shock protein*
(*Down*)					
Ug59429	AT5G54080.2	76.88	0	719	*Homogentisate 1*,*2-dioxygenase*
Ug103306	LOC_Os03g16880.1	80.91	1.00E-16	87	*Luminal-binding protein 4*
Ug104404	LOC_Os07g06890.1	78.10	1.00E-41	172	*D-lactate dehydrogenase*
Ug11935	LOC_Os06g46770.3	96.97	2.00E-07	56	*Ubiquitin-60S ribosomal protein*
Ug12499	AT5G03240.3	96.00	1.00E-16	82	*Ubiquitin-40S ribosomal protein*
**Ug130858**	Bg04-08_J10	78.49	6.00E-43	171	***Polyubiquitin 4***
Ug135016	LOC_Os01g65380.1	100.00	5.00E-06	52	*Chaperone protein dnaK*
Ug138511	LOC_Os08g31030.1	77.90	1.00E-39	163	*Protein HOTHEAD*
Ug2190	LOC_Os06g48650.3	77.74	3.00E-104	381	*Subtilisin-like protease*
**Ug22930**	LOC_Os10g40614.1	79.20	1.00E-14	82	***14 kDa proline-rich protein***
Ug27580	LOC_Os01g05790.1	73.77	1.00E-51	206	*Conserved hypothetical protein*
Ug47022	Bg01-06_P19	84.06	5.00E-51	198	*Gibberellin-regulated protein 4*
Ug59747	Bg04-20_N09	76.97	4.00E-80	296	*Abscisic acid receptor PYL8*
Ug77182	LOC_Os08g39140.3	73.78	0	641	*Heat shock protein 90-2*
Ug9466	LOC_Os08g43390.1	90.48	2.00E-06	54	*Cytochrome P450 78A3*
***Signaling*** (*Up*)					
Ug150075	LOC_Os08g37490.1	77.21	3.00E-24	111	*14-3-3-like protein GF14 kappa*
Ug154422	LOC_Os06g51170.1	74.57	3.00E-26	121	*Protein kinase APK1A*
Ug74868	LOC_Os05g25450.2	76.53	7.00E-36	152	*Receptor-like protein kinase*
(*Down*)					
Ug119376	LOC_Os04g43490.2	74.95	4.00E-59	230	*Casein kinase I isoform delta-like*
Ug121629	Bg05-07_K16	90.16	6.00E-15	80.5	*Probable LRR receptor-like serine/threonine-protein kinase*
Ug24897	LOC_Os03g20370.1	83.06	6.00E-73	274	*Calmodulin*
Ug55273	AT5G42440.1	85.29	1.00E-22	106	*Leucine-rich repeat receptor protein kinase*
Ug63319	Bg01-06_P19	79.37	9.00E-31	132	*Gibberellin-regulated protein 6*
***Transporters*** (*Up*)					
Ug40195	Bg03-06_L18	78.70	3.00E-46	185	*Organic Cation/carnitine transporter 7*
**Ug6107**	LOC_Os08g08070.1	70.21	7.00E-37	158	***Sugar carrier protein C***
Ug91442	Bg04-02_L05	77.62	3.00E-93	340	*Lysine histidine transporter 1*
Ug91704	LOC_Os04g55940.2	74.37	2.00E-113	411	*Vacuolar Cation/proton exchanger2 CAX2*
Ug29379	LOC_Os06g43660.3	79.69	3.00E-179	628	*Pyrophosphate-energized vacuolar membrane proton pump*
(*Down*)					
Ug86727	AT2G01420.2	93.75	2.00E-12	73	*Auxin efflux carrier component 2*
Ug138409	LOC_Os11g28610.1	77.89	6.00E-45	182	*Monosaccharide-sensing protein 2*
***Transcription-related*** (*Up*)					
**Ug113286**	LOC_Os04g51320.1	95.92	3.00E-14	80	***Trihelix transcription factor GT-3b***
**Ug42722**	LOC_Os05g34830.3	82.42	1.00E-56	222	***NAC domain-containing protein 2***
Ug42955	LOC_Os04g46250.1	79.09	2.00E-12	75	*Ethylene-response factor 1B*
Ug60441	LOC_Os02g06910.1	76.67	1.00E-33	145	*Auxin response factor 25*
(*Down*)					
Ug69295	Bg04-08_C14	83.44	6.00E-37	152	*Zinc finger CCCH domain-containing protein 69*
Ug29544	AT2G22840.1	84.25	2.00E-33	143	*Growth-regulating factor 6*
Ug124037	LOC_Os11g09690.1	96.97	2.00E-06	56	*Protein Mut11*
Ug129600	LOC_Os06g06510.1	77.80	2.00E-63	243	*Histone H3*.*2*
Ug39187	LOC_Os06g06510.1	78.76	5.00E-70	265	*Histone H3*.*2*
***Cytoskeleton- and trafficking-related*** (*Up*)					
Ug147417	LOC_Os12g44350.1	79.80	7.00E-35	147	*Actin-58*
Ug152164	LOC_Os09g39500.1	86.38	5.00E-60	231	*Ubiquitin-60S ribosomal protein*
**Ug78218**	LOC_Os01g18050.1	81.49	0	741	***Tubulin beta-8 chain***
Ug124903	LOC_Os03g58840.1	76.28	1.00E-81	305	*Vesicle-associated membrane protein 725*
Ug26019	AT1G10290.1	77.08	1.00E-127	455	*Dynamin-2A*
(*Down*)					
Ug10821	LOC_Os03g50885.1	79.74	0	773	*Actin*
Ug129620	LOC_Os03g51600.1	79.44	1.00E-64	246	*Tubulin alpha chain*
Ug130905	LOC_Os12g06660.1	83.36	7.00E-143	507	*Actin-1*
Ug130925	LOC_Os03g61970.1	77.65	2.00E-52	206	*Actin-1*
**Ug135408**	LOC_Os03g50885.1	85.30	4.00E-130	464	***Actin***
Ug139265	LOC_Os02g07060.1	83.49	3.00E-77	289	*Tubulin beta-7 chain*
Ug47023	Bg01-06_P19	86.67	3.00E-35	147	*Protein GAST1*
***Uncharacterized*** (*Up*)					
Ug44132	LOC_Os02g13970.2	74.70	5.00E-129	462	*Probable complex I intermediate-associated protein 30*
(*Down*)					
Ug45471	Bg04-21_O09	74.38	2.00E-44	180	*Uncharacterized protein*

Column 1 shows the unigene ID, while column 2 represents the ID of the reference gene. Percent similarity between the sequences of *A*. *officinalis* and the reference plant is shown in column 3 while e-value is given in the column 4. Column 5 indicates the bit score and the gene name is given in the column 6. The genes that were present in more than one data set are highlighted in bold.

### Mechanism of salt tolerance in *A*. *officinalis*

**Figure 2:**
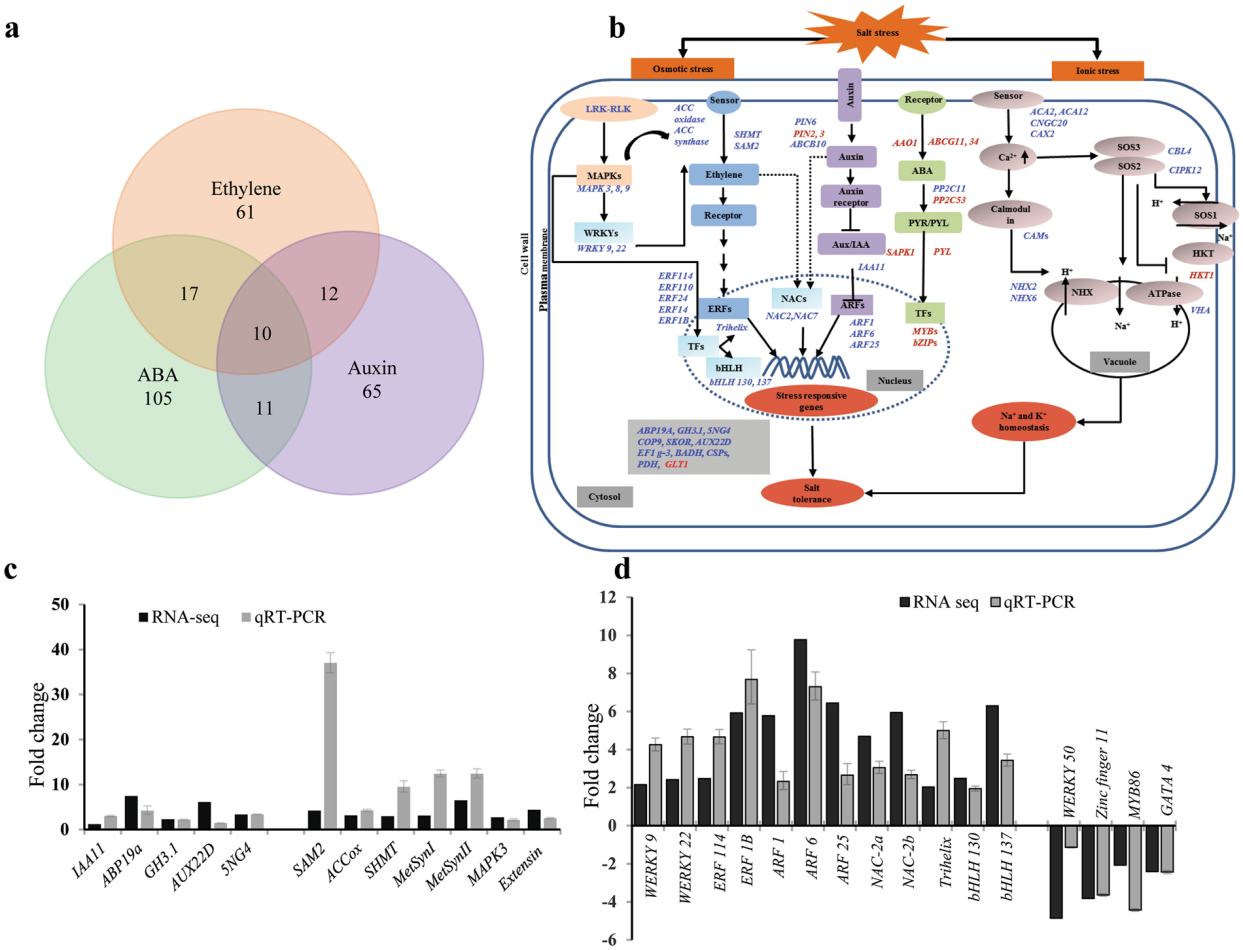
Phytohormone (ethylene and auxin) signaling-related genes are up-regulated upon salt treatment in *A*. *officinalis*. (**a**) Venn diagram represents the number ABA-, ethylene- and auxin-responsive DEGs identified in *A*. *officinalis* transcriptome analysis. (**b**) Signaling pathways mediating salt tolerance in *A*. *officinalis* roots: Major phytohormone (auxin, ethylene and ABA) and Ca^2+^ signaling pathways that are operative in various plants to render salt tolerance are depicted in the picture. Genes that are up-regulated in *A*. *officinalis* roots are indicated in blue, while the down-regulated genes are indicated in red. (**c**) Expression pattern of some of the genes related to ethylene- and auxin-signaling and (**d**) expression analysis of DEGs predicted to be encoding transcription factors. Black bar indicates transcript abundance changes calculated by RPKM method. The grey bars plotted with error bars represent the relative expression levels quantified by qRT-PCR method. Relative expression levels of transcripts with reference to *Ubiquitin 10* transcript levels are plotted, qRT-PCR data represent means ± SD, from 3 biological replicates. *IAA11*: *Auxin-responsive protein11*, *ABP19a*: *Auxin-binding protein*, *GH3*.*1: Probable indole-3-acetic acid-amido synthetase*, *AUX22D*: *Auxin-induced protein 22D*, *5NG4*: *Auxin-induced protein 5NG4*, *SAM2*: *S-adenosylmethionine synthase 2*, *ACCox*: *1-aminocyclopropane-1-carboxylate oxidase homolog 1*, *SHMT*: Serine *hydroxymethyltransferase*, *Metsyn*: *5-methyltetrahydropteroyltriglutamate–homocysteine methyltransferase*, *MAPK3*: *Mitogen-activated protein kinase3*.

Mere identification of candidate salt tolerance-related genes in the roots of *A*. *officinalis* is not sufficient to understand the broad regulatory network that involves the functioning of these gene products in rendering salt tolerance. We reasoned that phytohormone signaling, Ca^2+^ signaling and specific TFs should play important roles under salt stress to regulate many signaling pathways. Hence, all the identified DEGs that are predicted to be involved/associated with ABA, auxin and ethylene signaling pathways were analyzed in more detail, with the idea that they might reveal important signaling modules for mediating salt tolerance. In total, ~100 unigenes were ABA responsive while 65 and 61 were responsive to auxin and ethylene, respectively. While 11 of these genes were common to ABA and Auxin, 12 were common to auxin and ethylene and 17 were common to ethylene and ABA (Fig. 2a). Finally, 10 genes were found to be common in all the three pathways. In order to understand the potential roles of these genes in salt tolerance of *A*. *officinalis*, a broad signaling-network was created using the published information regarding these pathways^15, 53^. Upon placing the DEGs in these known pathways, it was evident that most of the DEGs involved in ethylene, auxin and Ca^2+^ signaling were up-regulated while those involved in ABA signaling were down-regulated (Fig. 2b). These results imply that several ABA-independent signaling pathways could also play a major role in salt tolerance of *A*. *officinalis*. Hence, the expression profiles of most of these genes were validated by temporal gene expression analysis using qRT-PCR (Figs S4 and S5).

#### Role of LRR-RLK and phytohormone signaling

A number of leucine-rich repeat receptor-like kinase (LRR-RLK) genes were up-regulated in the salt-treated roots, suggesting their possible role in perception of the stress signals. Although, the exact function of many LRR-RLK genes in plants have not been understood yet, RLKs are shown to be involved in cell to cell signaling under various environmental stresses by functioning as receptors to various signals^54^. Like other RLKs, LRR-RLK could be involved in phosphorylation of MAPKs, which is supported by the up-regulation of different MAPKs (MAPK3, 8 and 9) in our study (Fig. 2b). Moreover, expression of several genes involved in ethylene biosynthesis such as *methionine synthase*, *S-Adenosyl methionine synthetase* (*SAM2*), *SHMT* and *ACC oxidase* were observed to be upregulated in response to salt treatment in both RNA-seq and qRT-PCR experiments (Fig. 2c). In addition, *Trihelix* TF which is known to interact with AP2/ERFs^49^, *hexokinase1* known to be involved in ethylene signaling^55^, *cationic peroxidase* and *glutamate synthase* that are induced by ethylene leading to proline synthesis under salt stress^56–58^ are all identified as key salt tolerance-related genes (Table 4). Therefore, we hypothesize that ethylene signaling could be playing a major role in salt tolerance of mangrove roots. This hypothesis is further supported by the up-regulation of a number of AP2/ERF TFs such as *ERF 1B*, *14*, *24*, *110* and *114* (Fig. 2b). The expression of some of these *ERF*s was also confirmed by qRT-PCR analysis (Fig. 2d).

**Figure 3:**
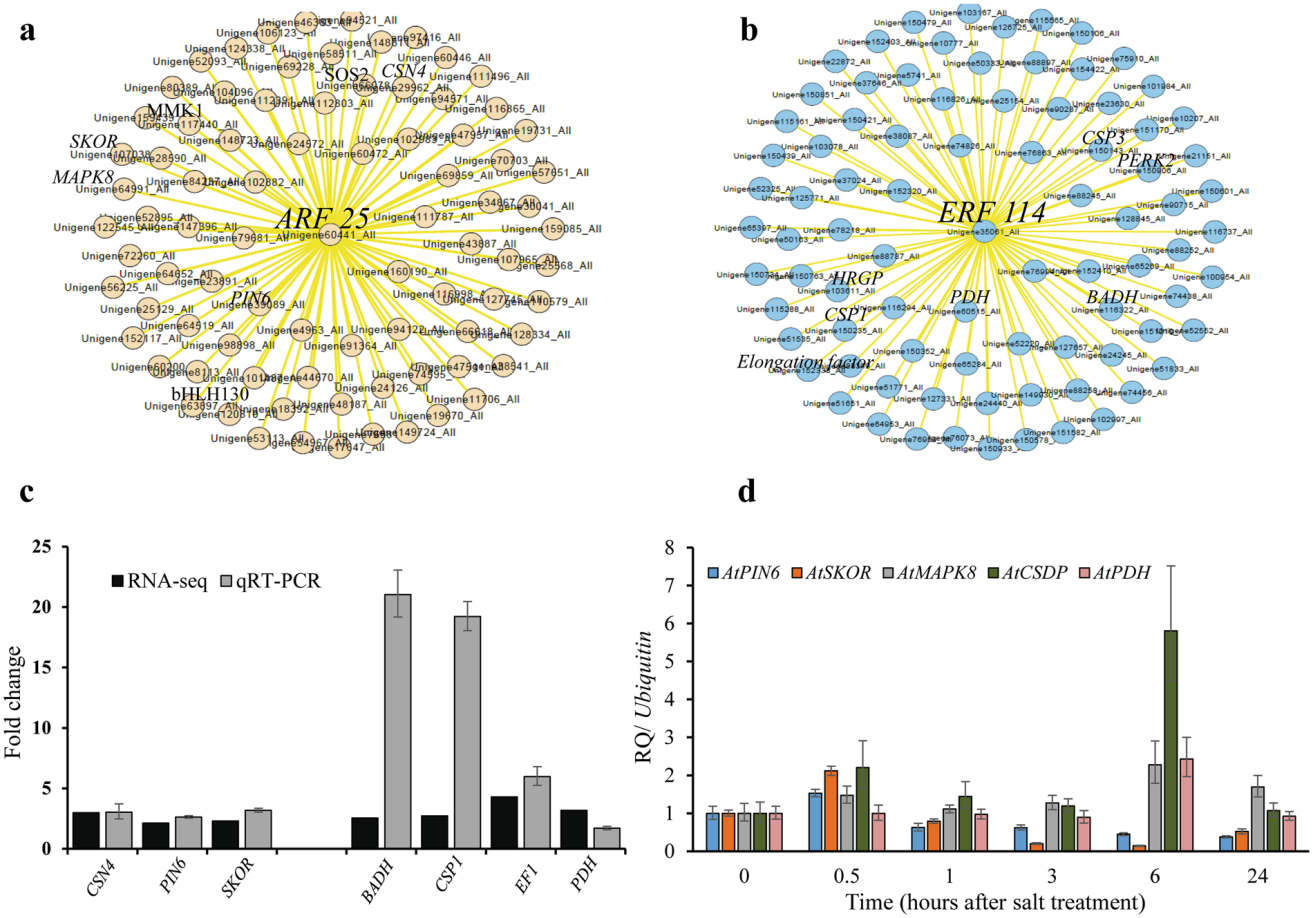
Gene network analysis using ARACNE and CYTOSCAPE. (**a**) Gene network analysis for *AoARF25* and (**b**) *AoERF114* is shown. Differentially expressed up-regulated genes were extracted from the RNA sequencing data and gene networks for selected genes were constructed using Algorithm for the Reconstruction of Accurate Cellular Networks (ARACNE) algorithm. Based on ARACNE output, the final gene network graphs were created using Cytoscape. Highlighted in black color in each network are the names of genes that are known to be involved in respective signaling pathways. The validation of the expression profile of a few of the selected genes by qRT-PCR analysis is shown in (**c**) *A*. *officinalis* roots and (**d**) *Arabidopsis* roots. Relative expression levels of transcripts with reference to *Ubiquitin 10* transcript levels are plotted, qRT-PCR data represent means ± SD, from 3 biological replicates. *CSN4*: *COP9 signalosome complex subunit 4*, *PIN6*: *PIN-FORMED6*, *SKOR*: *stelar K* ^+^ *outward rectifying channel*, *BADH*: *betaine aldehyde dehydrogenase*, *CSP1*: *cold shock protein1*, *EF1*: *elongation factor 1-gamma 3*, PDH: *pyruvate dehydrogenase*, *MAPK8*: *mitogen activated kinase8*, *CSDP*: *cold shock domain containing protein*.

Auxin [indole-3-acetic acid (IAA)] is essential for plant growth and development. It provides key signal for the formation of lateral roots in many plants. It is produced via tryptophan-dependent and -independent biosynthetic pathways and maintains its homeostasis by processes such as degradation, conjugation to amino acids and directional transport^59, 60^. In the current study, a number of auxin responsive genes such as probable *indole*-*3-acetic acid*-*amido synthetase GH3*.*1*, *auxin-induced protein 5NG4*, *ARF*s (6, 25 and 1) *auxin*-*binding protein ABP19a*, *auxin-responsive protein IAA11*, *auxin-induced protein AUX22D*, Auxin transporter *ABCB10*, probable auxin efflux carrier *PIN6*, *14-3-3* and *stelar potassium outward rectifying channel SKOR* were up-regulated (Figs 2c and 3c), strongly suggesting involvement of auxin signaling in the roots of *A*. *officinalis* in response to salt treatment. In further support of this hypothesis, *ARF25*, *PIN6* and *14-3-3* were also identified as the key salt tolerance-related genes by comparative genomic analysis (Table 4).

#### Role of transcription factors

In addition to the up-regulation of *ARF*s and *ERF*s, a number of TFs such as *NAC2*, *NAC7*, *WRKY*s (9 and 22), *bHLH*s (130 and 137) and *Trihelix* were also upregulated in *A*. *officinalis* roots (Fig. 2d and Table 2). Among these TFs, NAC2/NAC7 could be common downstream components of both auxin and ethylene signaling pathways. The induction of these TFs could be acting in an ABA-independent manner to support lateral root development^61^. Overall, our results suggest a potential involvement of a number of TFs and a crosstalk between auxin and ethylene signaling in response to salt treatment. This is further supported by down-regulation of a number of genes involved upstream and downstream of ABA signaling pathway and many ABA responsive TFs such as *MYB*s, *ABF*s and *bZIP*s. Our results also provide possible link between upregulation of *WRKY 9* and *WRKY 22* TFs and activation of genes for induction of ethylene through regulation of ACC synthase activity. Although, ACC synthase activity is known to be regulated by MAPK3/MAPK6 cascade and the downstream WRKY TF during ethylene production^62^, the MAPK cascades involved in phosphorylating WRKYs have not been well studied under abiotic stress compared to biotic stress. However, up-regulation of *WRKY9* and *WRKY22* along with *MAPK*s (3, 8 and 9) together suggests their possible key role in inducing ethylene biosynthesis and signaling. Involvement of bHLH TF in salt stress response has been shown in a few plant species^63^ and induction of both bHLHs (130 and 137) and different peroxidase (POD) genes in our study suggests their possible positive involvement in the regulation of peroxidase-mediated reactive oxygen species removal during salt treatment in *A*. *officinalis*. The Trihelix TF, could also play an important role in stress signaling because this TF has been identified as a key salt tolerance-related gene (Table 4) and was highly induced by salt in *Arabidopsis* ^49^.

The association of hormones and different TFs was further evident in the interaction network created using ARACNE and CYTOSCAPE to identify important stress-responsive pathways (Fig. 3a and b). Among different potential genes, the *AoARF25* and *AoERF114* were preferred as specific nodes due to their significant upregulation in response to salt treatment in the roots of *A*. *officinalis*. In addition, *AoARF25* was also identified as a salt tolerant gene by comparative genomic analysis (Table 4). The network analysis with *AoARF25* showed interactions with 76 up-regulated genes (Table S3) including *PIN6* (an auxin efflux carrier), *MAPK*s (*MAPK8* and *MMK1*), *COP9 signalosome complex subunit 4* (*CSN4*), *bHLH* TF, *serine/threonine-protein kinase* (*SOS2*) and potassium channel (*SKOR*). Whereas, *AoERF114* was possibly interacting with *proline*-*rich receptor*-*like protein kinase* (*PERK2*), *betaine aldehyde dehydrogenase* (*BADH*), *cold-shock proteins* (*CSP1*, *CSP3*), *extensin* (*HRGP*), *pyruvate dehydrogenase* (*PDH*) and *elongation factor 1-gamma 3*. To further validate the expression pattern of these genes, qRT-PCR analysis was performed in both *A*. *officinalis* (Fig. 3c) and *Arabidopsis* by profiling the expression of some of the homologous genes (Fig. 3d). Both the network and the gene expression results provide extensive information regarding the involvement of complex interactions of phytohormones in response to salt treatment in *A*. *officinalis*.

#### Role of Ca^2+^ signaling

Calcium is one of the most important second messengers required for plant signaling networks under abiotic stresses. Many external stimuli like salt stress are known to increase Ca^2+^ levels in the cytosol within seconds through various Ca^2+^ transporters and pumps^64^. In the current study, several genes related to Ca^2+^ signaling were up-regulated (Figs 2b and S5b). Calcium-transporting ATPases (*ACA*s), Ca^+^/H^+^ exchangers (*CAX*s) and *CNGC*s were differentially expressed, which could be leading to the Ca^2+^ fluxes during salt stress. The CNGC20 could be involved in Ca^2+^ influx across PM, while ACAs (ACA12, ACA2) and CAX2 could be involved in efflux across PM and tonoplast, respectively. The increased Ca^2+^ levels are sensed by the calcium sensors such as CaMs and CBLs (SOS3)^65^, both of which were up-regulated in the current study. Further, SOS3 interacts with specific Ser/Thr kinases (CIPKs/SOS2) and this SOS3-SOS2 complex would activate various downstream targets under salt stress^53, 64^. This complex would activate Na^+^/H^+^ antiporters SOS1 and NHX1, leading to Na^+^ efflux across PM and Na^+^ compartmentalization into vacuoles, respectively^53, 66^. They are also known to block Na^+^ uptake in the roots by HKT1, leading to salt tolerance in plants. Concomitantly, both *NHX1*and *NHX6* were up-regulated; whereas *HKT1* was down-regulated in our study. Although, a number of *SOS1* genes were identified, they were not differentially regulated with 24 h salt treatment. Also, the vacuolar ATPase, *VHA* was up-regulated, which has previously been shown to be activated by this complex. Identification of *CAX2* and *calmodulin* as salt tolerance-related genes (Table 4) makes their role even more significant in salt tolerance. Overall, our results suggest that in addition to phytohormones, Ca^2+^ signaling could play an important role in salt tolerance of *A*. *officinalis*. However, further experiments are required to confirm the roles of these identified candidate genes in salt tolerance of mangroves.

### Ethylene response factor (*AoERF114*) plays an important role in salt tolerance: a case study using *Arabidopsis* mutant *aterf115*

**Figure 4:**
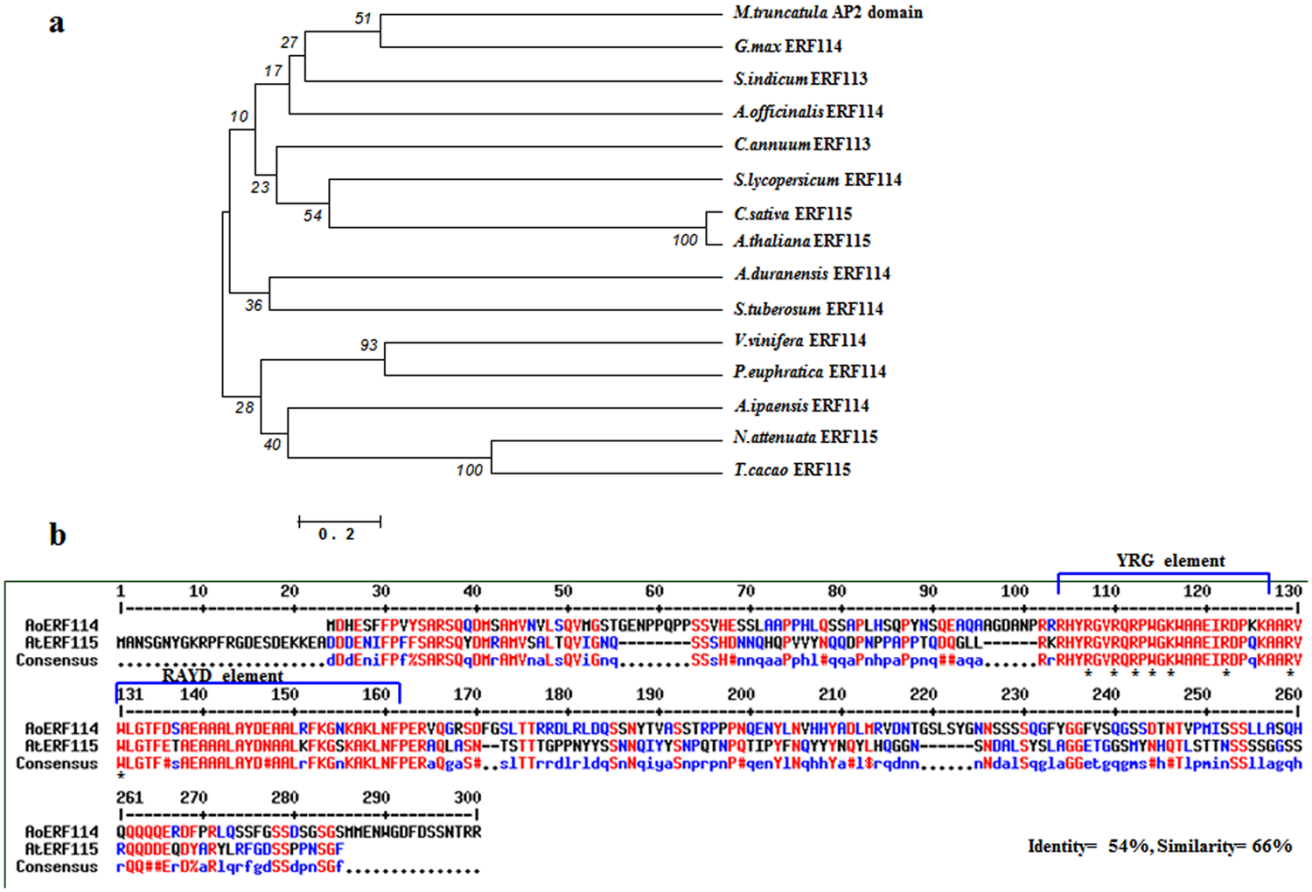
The AoERF114 is homologues to AtERF115. (**a**) Phylogenetic tree derived from deduced amino acid sequence alignment of ERF114/ERF115 from *Vitis vinifera* (XP_010663806.1), *Glycine max* (XP_003522453.1), *Populus euphratica* (XP_011043029.1), *Theobroma cacao* (EOY22656.1), *Camelina sativa* (XP_010491335.1), *Arabidopsis thaliana* (NP_196348.1), *Medicago truncatula* (XP_003602747.1), *Capsicum annuum* (XP_016563056.1), *Nicotiana attenuata* (OIT36443.1) *Solanum tuberosum* (XP_015163844.1), *Sesamum indicum* (XP_011073204.1), *Solanum lycopersicum* (XP_004252471.2), *Arachis ipaensis* (XP_016169154.1) and *Arachis duranensis* (XP_015937725.1). The phylogenetic tree was constructed using MEGA 6.0 with the neighbor-joining method, Poisson correction and bootstrap value of 500^110, 111^. The bootstrap branch support values are shown at the nodes and scale bar indicates the branch lengths. (**b**) Sequence alignment of the derived amino acid sequences of AoERF114 and AtERF115. The distinctive YRG and RAYD elements within the conserved AP2 domain are highlighted. The eight conserved amino acids involved in DNA contact are indicated by asterisks.

**Figure 5:**
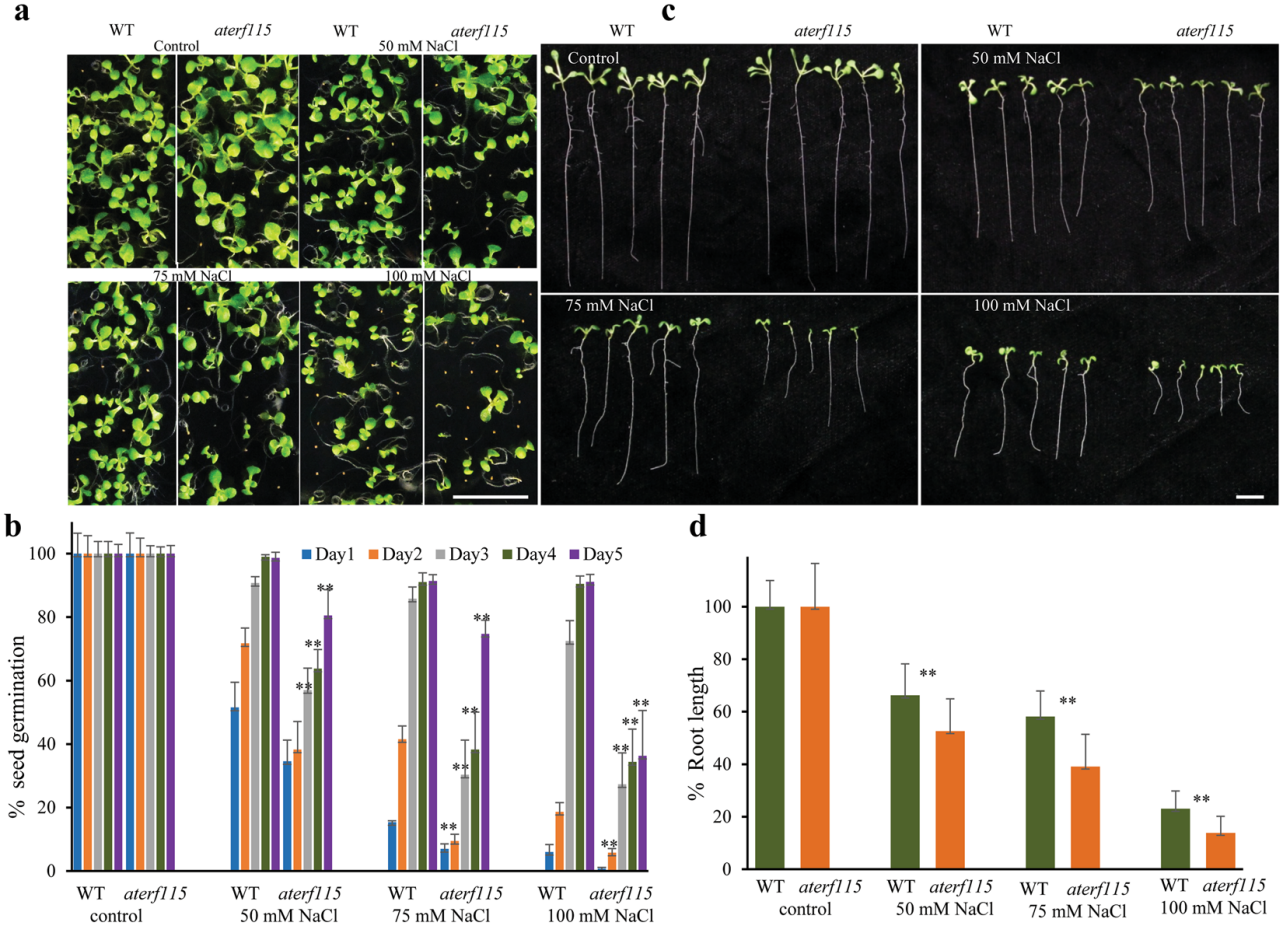
*Arabidopsis aterf115* seedlings are sensitive to salt. (**a**) Photographs and (**b**) bar graph showing the dosage dependent reduction in the germination of seeds of *aterf115* compared to wild type. Both WT (Columbia) and *aterf115* seeds were surface sterilized and cold stratified for 3 days before sowing onto MS agar plates containing NaCl (0–100 mM). The number of germinated seeds were counted from day 1 to 4 and the photographs were taken 7 days after germination. (**c**) Pictures depicting the salt sensitivity of the *aterf115* seedlings to salt treatment. (**d**) Graph showing the rate of root growth under varying external salt. Surface sterilized and cold stratified seeds were sown onto MS agar plates containing NaCl (0–100 mM). Photographs and root lengths were measured at the end of seven days after germination. Scale bar = 10 mm.

Integration of the gene expression data, network analysis and the validation results suggest the importance of ethylene signaling via ERFs in salt tolerance of *A*. *officinalis*. In our study, both *AoERF114* and *AoERF1B* were significantly up-regulated (Fig. 2d). Although, *ERF1B* was identified as a salt tolerance-related gene (Table 4), its expression was suppressed upon salt treatment in *Chrysanthemum* ^67^. Therefore, we chose *AoEFR114* for a more elaborate study. Based on the phylogenetic tree generated using the deduced amino acid sequence of *AoERF114* and other members of the family from the database, AtERF115 emerged to be one of its homologs in *Arabidopsis* (Fig. 4a). Sequence alignment of the derived amino acid sequences of AoERF114 and AtERF115 showed 54% identity and 66% similarity between the two. The AP2 domain characteristic of the AP2/ERFs consisting of YRG and RAYD elements^68^ was also conserved in both (Fig. 4b). Moreover, eight conserved amino acid residues that are involved in the interaction with the DNA GCC box^69^ are present in AoERF114 and AtERF115. The *Arabidopsis* homozygous insertional mutant *aterf115* (AT5G07310.1, SALK_021981 C) was obtained from TAIR to study the effect of salt treatment. The mutant was more sensitive to salt compared to wild-type (WT) seedlings (Fig. 5). Seed germination of *aterf115* was significantly reduced (more than half) upon 75 mM and 100 mM NaCl treatment (Fig. 5a and b). In addition, salt treatment significantly affected the seedling growth of *aterf115* on agar plates (Fig. 5c and d). Moreover, we found that the roots of *aterf115* seedlings were shorter than that of WT seedlings when treated with 75 mM and 100 mM NaCl (Fig. 5c).

**Figure 6:**
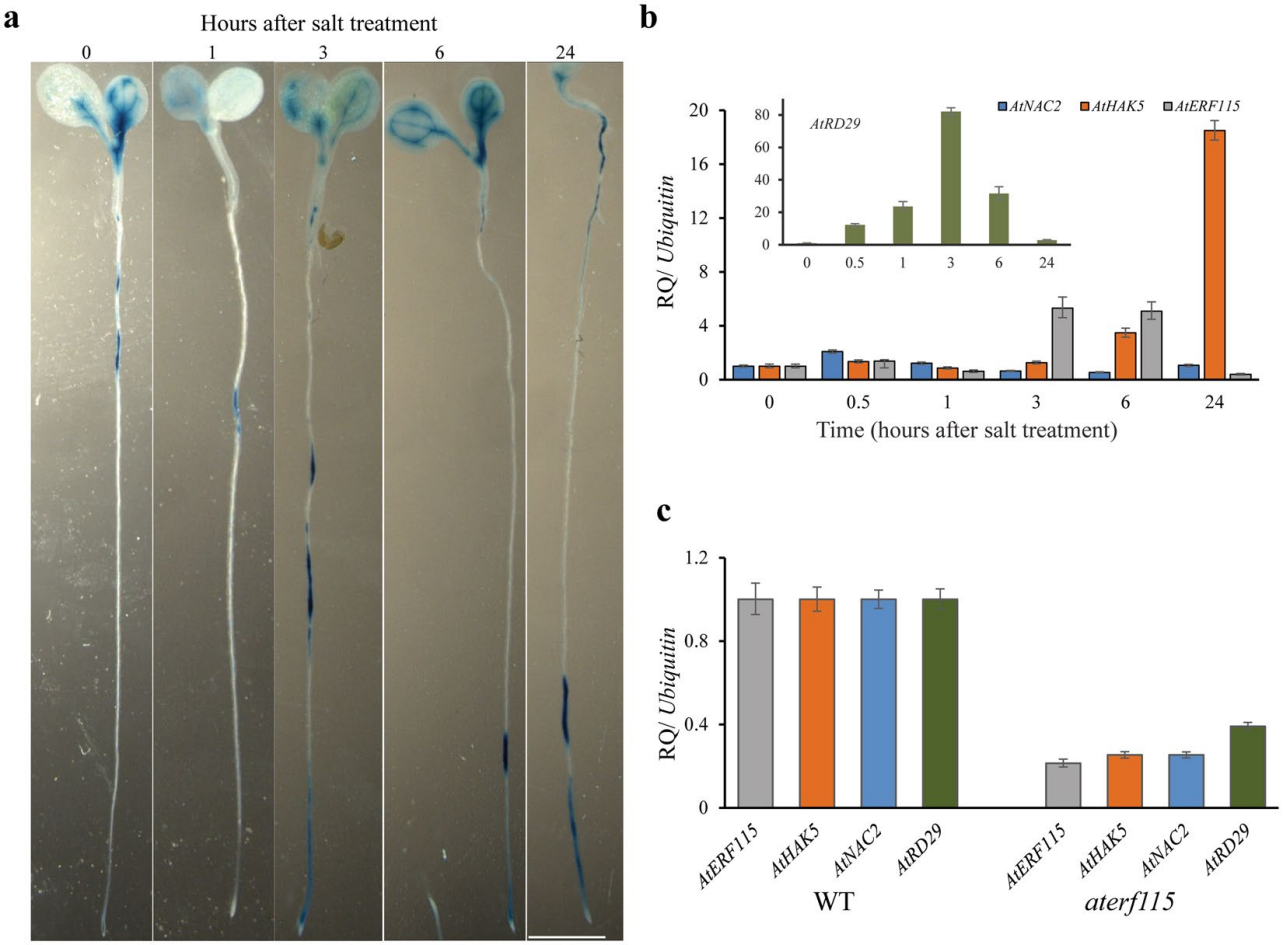
Transcripts of *AtERF115* are induced upon salt treatment. (**a**) Photographs showing induction of *AtERF115* transcript levels in seedlings of *pAtERF115*::GUS lines upon salt treatment. Surface sterilized seeds were cold stratified and sown onto MS agar plates. Five-day-old seedlings were treated with 50 mM NaCl for varying time periods (0–24 hours) and then stained with GUS and photographed as described in the Materials and Methods. Scale bar = 0.5 mm (**b**) Graph showing qRT-PCR analysis of temporal expression of *AtERF115* along with a few of the reported target genes of ethylene response factors under salt treatment (150 mM NaCl for varying time periods) in *Arabidopsis* roots. (**c**) Expression profile of a few of the reported target genes of ethylene response factors in the *aterf115* mutant roots by qRT-PCR. Relative expression levels of transcripts with reference to *Ubiquitin 10* transcript levels are plotted, qRT-PCR data represent means ± SD, from 3 biological replicates. *ERF115*- *ethylene response factor115*, *NAC2*- *No Apical Meristem domain-containing factor2*, *HAK5*- *high affinity potassium transporter5*, *RD29*- *responsive to desiccation29*.

In order to test whether *AtERF115* responds to salt treatment, we obtained *Arabidopsis* lines with GUS expression driven by the promoter of *AtERF115* (*pAtERF115::GUS* line). The GUS expression patterns in the roots showed that *AtERF115* gene was induced in response to 3 to 24 h of salt treatment (Fig. 6a). Similarly, the transcript levels of *AtERF115* increased upon salt treatment after 3 and 6 h as shown by qRT-PCR analysis (Fig. 6b). In addition, the expression profile of selected, known targets of ERFs were tested and were found to be significantly up-regulated by salt treatment in *Arabidopsis* roots (Fig. 6b). While *NAC2* showed a twofold increase (after 0.5 h of salt treatment), *HAK5* and *RD29* showed 18-fold (after 24 h) and 80-fold (after 3 h) increase, respectively. To independently verify this data, the expression profiles of these selected target genes were checked in the *aterf115* mutant seedling roots, and they were significantly reduced (Fig. 6c). Overall, these findings suggest that the ERF115 TF could be involved in ethylene signaling by regulating some of these genes.

## Discussion

In this study, a comprehensive transcriptomic analysis from the roots of *A*. *officinalis* in response to salt treatment was carried out in order to identify salt-responsive genes. Despite the advancement in genome sequencing techniques, genomic information for many non-model plants is unavailable. Transcriptome profiling using mRNA-sequencing facilitates rapid generation of large datasets leading to identification and quantification of transcripts even in the absence of a reference genome sequence^70^. While transcriptome studies for a few salt secretor mangrove species have been carried out^32–34^, such information on *A*. *officinalis* is missing.

In general, roots provide the first line of defense against salinity as they are in direct contact with the saline soil. This necessitates them to exhibit anatomical, physiological and molecular changes in order to adapt to such harsh environments. Therefore, the primary and important mechanisms of salt tolerance may reside in the roots. Salt tolerance is a complex phenomenon which involves the interaction of many genes that brings about tissue tolerance to osmotic stress, ion homeostasis and detoxification^1^. In the current study, several groups of potential salt-responsive genes, which could contribute to the salt tolerance of *A*. *officinalis*, were identified. Some of the important salt-related genes are discussed in relation to their known functions from other plant species.

Mangroves have been shown to accumulate high levels of organic solutes such as proline, glycinebetaine, polyols and sugars in order to overcome the salinity-induced osmotic stress^5^. In support of this, among the group of genes that affect metabolites, we observed significant up-regulation of *choline monooxygenase* (*CMO*) and *BADH* involved in glycinebetaine biosynthesis^71^ as well as *Hexokinase1* (*HXK1*) and *trehalose 6-phosphate phosphatase* (*TPPA*) involved in trehalose biosynthesis^72^ in *A*. *officinalis* roots treated with salt. *BADH* and *CMO* have also been reported from various species like *Suaeda*, *Halogeton*, *Atriplex*, *sugar beet etc*.^22, 25, 73, 74^. We also observed up-regulation of several key genes encoding enzymes that are involved in reactive oxygen species (ROS) scavenging and detoxification under salt stress^75, 76^. These include peroxidases, catalases, glutathione peroxidases and glyoxylases. These ROS scavenging enzymes are well studied in plants and are known to be associated with various abiotic stresses. Additionally, genes related to flavonoid biosynthesis were also up-regulated in our study. Flavonoids have been shown to enhance salt tolerance by mitigating oxidative damage in soybean^77^. Up-regulation of these genes suggests that the oxidative stress is induced by salt treatment, and detoxifying as well as ROS scavenging enzymes are active in the mangrove roots as part of the metabolic adaptation towards salt tolerance as seen in other halophytes^5, 74^.

Another major group of DEGs identified in our study comprised of genes involved in regulating ion uptake and transport. Roots have remarkable ability to regulate the plant’s Na^+^ and Cl^−^ concentrations. Among the genes known to confer salt tolerance are those that are associated with ion uptake, transport to shoots, root ion homeostasis and water status^78^. Non-selective cation channels such as CNGCs are known to be involved in uptake of Na^+^, K^+^ and Ca^2+^ ^79^ while NHXs are involved in Na^+^, K^+^ compartmentalization and pH homeostasis, which function by utilizing the pH gradient generated by V-ATPases^80^. K^+^ transporters are essential to maintain the ionic balance which is altered under salt stress^81^. In *A*. *officinalis*, several plasma membrane and tonoplast transporters were up-regulated (Table S2). The CNGC could be involved in ion uptake while NHXs along with V-ATPases and K^+^ transporters could function in ion sequestration and homeostasis. The precise function of ABC transporters is still unknown. However, they are implicated in various functions including transport of heavy metals, osmolyte, fatty acids, auxin and Na^+^^82–84^. Overall, the data suggest that various genes involved in osmotic adjustment, ion homeostasis, detoxification and metabolic processes are up-regulated and could play important roles in salt tolerance of *A*. *officinalis*. However, the functionality of these genes needs further experimental validation.

With the aid of comparative genomic approach, key salt tolerance-related genes in *A*. *officinalis* roots were identified, while the temporal expression profiles of a few of them were validated by qRT-PCR. Presence of some of these genes (13) in more than one dataset strengthens their importance in salt tolerance of plants. Among these genes, *Glutamate synthase1* was shown to be involved in proline synthesis under salt stress in tomato and its activity was increased by ethylene in *Hevea* leaves^57, 58^. Hexokinase1 plays important role in sugar and ethylene signaling^31, 55^. These findings suggest that both metabolism-related genes could be involved in accumulation of osmolytes required for osmotic balance under salt stress in *A*. *officinalis* along with the other genes discussed earlier. Interestingly, among the four classes of TFs identified (*Trihelix*, *NAC*, *ARF*, *ERF*), *Trihelix* and *NAC* were present in more than one dataset and hence they can be important candidates for future studies in understanding salt tolerance mechanisms in plants. *NAC*s and *Trihelix* TFs were also found to be differentially regulated by salt in *Suaeda maritima* ^22^.

Although we have identified 93 genes as salt tolerance-related genes, the relevance and importance of other DEGs cannot be ignored. The changes in expression may not be the same across various species compared, because they may have different mechanisms of response, involving different molecular elements. Even if the same stress treatment is applied, it is expected that two plant species may experience different levels of stress, and accumulate a given transcript at different levels. Considering the broad diversity of salt tolerance mechanisms in plants, diverse gene expression profiles under salt treatment is not unusual. Hence, it will be important to study all the genes that are responsive to salt treatment in our attempts to unravel the tolerance mechanism.

The results based on gene network and signaling network analyses suggest that there is a crosstalk between auxin and ethylene signaling in response to salt treatment, which may operate in an ABA-independent pathway in *A*. *officinalis*. This is further supported by the observation that many genes known to be upstream and downstream of ABA signaling pathway were down-regulated (Fig. 2b). Moreover, many ABA dependent TFs such as *MYB*s, *ABF*s and *bZIP*s were also down-regulated (Table 2). In addition to salt and osmotic stresses, mangrove roots regularly experience submergence stress, which requires them to adapt to hypoxic conditions. Ethylene biosynthesis has been shown to be increased in roots under hypoxic condition and involvement of ethylene in inducing aerenchyma formation has also been well studied in other plant systems^85, 86^. This explains the up-regulation of several AP2/ERF transcription factors and genes involved in ethylene signaling in *A*. *officinalis*. This TF either alone or in combination with other ERFs and TF families could be involved in the control and regulation of ROS accumulation and signaling that is required for adaptation to salt and sub-ambient oxygen concentration. Both higher salt levels and waterlogging inhibit root elongation^87^. While there is ion toxicity to the roots in the former, there is reduced oxygen supply in the latter. Furthermore, studies indicate that the stress hormone ethylene, which accumulates in waterlogged plants, can contribute to the regulation of lateral and adventitious root formation in a complex crosstalk with auxin^88^. Many NAC TFs are found to be responsive to auxin, ABA and abiotic stresses in *Arabidopsis* ^61^. Similarly, we hypothesize that transcription factors NAC2/NAC7 could be promoting lateral root formation under submerged conditions in coordination with auxin and ethylene signaling pathways in the mangrove system reported here. In addition, it was shown that NAC2 in *A*.*thaliana* promoted lateral root formation and salt-induced *AtNAC2* expression was dependent upon the ethylene and auxin signaling pathways, but not ABA signaling^61^. Under salt stress, ethylene signaling in plants is known to be mediated through ERFs which regulate the stress responsive genes^89^. Some of the salt responsive genes regulated by ethylene are: A dehydration responsive gene *RD29* ^90^, *NAC2* ^61^ and high affinity potassium transporter (*HAK5*)^91, 92^. Our results suggest that *AoERF114*/*AtERF115* could be regulating some of these genes to render salt tolerance as all of these genes were suppressed in the salt sensitive *aterf115* roots, while their expressions were induced upon salt treatment in the WT (Fig. 6b and c). Therefore, further studies on ERFs (especially ERF115) are necessary to unravel the mechanism of stress response in plants. In addition to phytohormones, Ca^2+^ signaling is well known to play important role in salt tolerance of plants^93^. Up-regulation of several genes involved in this signaling pathway, underlines their importance in *A*. *officinalis*. The NHXs (NHX2 and NHX6) and vacuolar ATPases could be important for Na^+^/K^+^ homeostasis in *A*. *officinalis* roots, similar to their function in other plants^94, 95^. NHX1 and V-ATPases from several halophytes have proven to increase salt tolerance in glycophytes^25^. However, NHXs were not responsive to salt treatment in the leaves of halophytes like *Suaeda* and *Halogeton* ^22, 74^. Overall, identification of a number of up-regulated genes associated with ethylene, auxin as well as Ca^2+^ signaling provides critical information regarding the involvement of these signaling pathways in salt tolerance of mangroves.

In conclusion, a comprehensive transcriptome profile of *A*. *officinalis* roots is provided in this study. Our data helped to identify numerous salt tolerance-related genes as part of an overall list of DEGs in response to salt treatment, 93 of which would conceivably be playing meaningful roles in conferring salt tolerance in mangroves and other plants. The transcriptome data together with our results from *Arabidopsis* mutant (*aterf115*) analysis helped to reveal an important role for this ERF in salt tolerance. Our study also revealed the interplay of various *A*. *officinalis* genes involved in ethylene-, auxin- and Ca^2+^-mediated signaling pathways in a salt-responsive manner. This information may be used for future studies on salt tolerance in plants.

## Materials and Methods

### Plant materials and growth conditions (*A*. *officinalis*)

The propagules of *Avicennia officinalis* L. (*A*. *officinalis*) were collected during fruiting seasons from the mangrove swamps in Singapore (Berlayer Creek and Sungei Buloh Wetland Reserve). The seedlings were maintained in NaCl-free conditions by growing in potting mixture (Far East Flora, Singapore), until they reached the four-node stage (~2 months) in a greenhouse (25–35 °C, 60–90% relative humidity; 12 h photoperiod), after which they were carefully transferred to pots containing sand and were allowed to adapt for two days by watering with half-strength Hoagland’s solution. The plants were then treated with half-strength Hoagland’s solution containing 500 mM NaCl for 24 hours.

### Plant materials and growth conditions (*Arabidopsis*)

Wild-type *Arabidopsis* (*Arabidopsis thaliana*, ecotype Columbia), *aterf115* mutant and *pAtERF115::GUS* ^96^ lines were used in this study. The *aterf115* was supplied by the Arabidopsis Biological Resource Center of Ohio State University (Columbus, OH, USA). After sterilization and cold stratification at 4 °C for 3 days, the seeds were sown on MS agar plate and germinated at 22 °C under continuous light. The 10-day-old seedlings were carefully removed from the plate and subjected to salt treatment with 150 mM NaCl. The plants were collected at various time periods (0 h, 0.5 h, 1 h, 3 h, 6 h and 24 h) and frozen in liquid nitrogen for total RNA isolation. For histochemical GUS expression analysis, 5-day old seedlings were treated with 50 mM NaCl for various time periods (0 h, 1 h, 3 h, 6 h and 24 h). For seed germination studies, the sterilized and cold stratified seeds were sown on MS Agar plate with and without NaCl and allowed to germinate as mentioned above. The number of germinated seeds was counted from day 1 to day 4 and the root lengths were measured and photographed 7 days after germination.

### RNA isolation

Total RNA was isolated from roots of control and treated (500 mM NaCl for 24 h) greenhouse-grown *A*. *officinalis* using Qiagen RNeasy kit (QIAGEN) and DNase treated (RNase-free DNase set, QIAGEN) according to the manufacturer’s instructions. The quality of RNA samples was determined using a 2100 Bioanalyzer (Agilent Technologies). For each sample, at least 20 µg of total RNA was sent to Beijing Genomics Institute for Illumina sequencing (commercial service). For qRT-PCR experiments, total RNA was isolated from the roots of control and treated (500 mM NaCl for varying time periods; 0 h, 0.5 h, 1 h, 2 h, 4 h, 8 h, 12 h and 24 h) greenhouse-grown *A*. *officinalis* and control and treated (150 mM NaCl for varying time periods; 0 h, 1 h, 3 h, 6 h and 24 h) roots of one-week-old *Arabidopsis* seedlings as described above. An aliquot of this RNA (1 µg) was used to synthesize cDNA using Maxima first strand cDNA synthesis kit for qRT-PCR (Thermo Scientific) following manufacturer’s instructions.

### cDNA library preparation, sequencing and transcriptome *de novo* assembly

For each sample, mRNAs were purified using oligo (dT)-attached magnetic beads and fragmented into small pieces (100–400 bp). The cDNA library was prepared by synthesizing the first and second strand cDNAs, using the mRNA fragments as templates primed with random hexamers. The synthesized cDNAs were end repaired, 3’ adenylated and ligated with sequencing adaptors. Suitable fragments (~200 bp) were selected by agarose gel electrophoresis and enriched by PCR amplification. Finally, these cDNA libraries were sequenced using Illumina HiSeq™ 2000 sequencer (Beijing Genomics Institute, BGI, Shenzhen, Guangdong, China). Image data obtained from the sequencing machine was transformed by base calling into sequence data (raw reads) and stored in fastq format. Transcriptome *de novo* assembly was performed using the short read program Trinity (version release-20121005)^97^. The Trinity software first combined clean reads with a specific length of overlap to form longer fragments without Ns, forming contigs. Next, the contigs were connected to obtain consensus sequences that contained the least Ns and could not be extended on either end. Such sequences were defined as unigenes. Finally, the sequence orientations of the all-unigenes were determined by Blastx against NCBI non-redundant (Nr) protein database, Swiss-Prot, Kyoto Encyclopedia of Genes and Genomes (KEGG), and Clusters of Orthologous Groups (COG) with e-value cut off of <10^−5^. Unigenes that could not be aligned to any of the four databases were scanned using EST Scan^98^, which produced a nucleotide sequence (5′–3′) direction and amino sequence of the predicted coding region. The transcriptome data of this work has been deposited to the NCBI website (GEO GSE73807).

### Data analysis

For functional annotation, unigene sequences were first aligned using Blastx to the Nr, Swiss-Prot, KEGG, and COG protein databases (E-value < 10^−5^), which retrieved proteins with the highest sequence similarity to *A*. *officinalis* unigenes in addition to their protein functional annotations. Sequence searches were performed by querying the NCBI Nr protein database using the Blastx algorithm (E-value < 10^−5^)^99^. After Nr annotation, the Blast2GO program^100^ was used to obtain Gene Ontology (GO) annotations and the WEGO software^101^ was used to perform GO functional classification of all unigenes to determine the distribution of gene functions at the macro level. KEGG annotation was carried out to obtain pathway annotations for unigenes. Later, unigenes were aligned to the COG database to predict and classify potential functions based on known orthologous gene products using pathfinder software (version release 63.0). Gene expression analysis was carried out using reads per kilobase per million reads (RPKM) method^102^. For a given unigene, RPKM values were generated using SOAP (version release 2.21). A rigorous algorithm was used to identify differentially expressed genes (DEGs) in salt-treated roots compared to untreated roots. False discovery rate (FDR) ≤ 0.001, the absolute value of log2Ratio ≥2 and P-value ≤ 0.001 was used as the threshold to judge the significance of differential gene expression^103^. For pathway and GO enrichment analysis, all DEGs were mapped to KEGG and GO databases (http://www.geneontology.org/). By using hypergeometric test, significantly enriched GO terms were identified in comparison with the genome background. In addition, the DEGs were classified into various GO categories, based on the published databases and reports on particular genes. To identify important salt tolerance-related genes, the sequences of the DEGs were aligned with the published transcriptome/microarray sequences obtained in response to salt treatment from roots of *Arabidopsis*, rice and a mangrove species *Bruguiera*. The main criteria for choosing these species was that the transcriptomic/microarray sequences were obtained in response to salt treatment from the roots of the plants. The commonality among the various datasets (from the different plant species) used was that they were all “salt responsive datasets from roots”. From *Arabidopsis*, two published datasets were used^104, 105^ with the GEO IDs; GDS3216 and GSE46208. Similarly, two published datasets were used from rice^106, 107^ with the corresponding GEO IDs; GSE20746 and GSE14403 and one published data with the GEO ID GSE10942 was used from *Bruguiera gymnorhiza* ^31^.

### Histochemical GUS staining

Transgenic *Arabidopsis* seedlings containing *pAtERF115::GUS* fusion constructs were treated as described above. GUS histochemical staining was performed by vacuum-infiltrating the seedlings immersed in GUS staining solution (0.1 M sodium phosphate buffer pH 7.0, 10 mM EDTA, 0.1% Triton-X, 2 mM 5-bromo-4-chloro-3-indolyl glucuronide (X-Gluc)) for 5 min followed by overnight incubation in the dark at 37 °C without shaking. Staining solution was removed and several washes with 50% ethanol was performed until the chlorophyll was bleached and tissues cleared. The image of blue colored whole seedlings with various salt treatments was recorded using a stereo microscope (Leica DIC 310 FX). GUS- stained tissues and plants shown in this paper represent the typical results of at least six independent plants for each treatment.

### Quantitative real-time PCR (qRT-PCR) analysis

The qRT-PCR for differentially expressed genes was performed using the Stepone Real-Time PCR machine (Applied Biosystems) with the following programme: 20 s at 95 °C followed by 40 cycles of 03 s at 95 °C and 30 s at 60 °C. The SYBR Fast ABI Prism PCR kit from KAPA was used for qPCR analysis. The reaction mixture consisted of 5.2 μL master mix (provided in the kit), 0.2 μM FW primer, 0.2 μM RV primer, 3.4 μL nuclease-free water, and 1 μL sample cDNA template for a final volume of 10 μL. All of the data were analyzed using the StepOne^*TM*^ Software (v2.1, ABI). The primers were designed using the sequences obtained by RNA sequencing and are listed in Supplemental Table S4. Constitutively expressed *Ubiquitin 10* was used as internal control.

### Network analysis

Differentially expressed up- or down-regulated genes were extracted from the RNA sequencing data of the root samples of *A*. *officinalis*. Gene networks for selected genes were constructed using Algorithm for the Reconstruction of Accurate Cellular Networks (ARACNE) algorithm^108^. ARACNE uses the mutual information of the features to determine the connection between genes. The features included in these networks were gene expression (RPKM) and transcript-to-SWISPROT protein alignment score, among others. Based on ARACNE output, the final gene network graphs were created using Cytoscape^109^.

## Electronic supplementary material

Supplementary material
