# Supplementary material for: Transcriptomics analysis of salt stress tolerance in the roots of the mangrove Avicennia officinalis
Source: Sci Rep. 2017 Aug 30;7:10031. doi: 10.1038/s41598-017-10730-2 (PMC5577154; doi:10.1038/s41598-017-10730-2)
Supplement: Supplementary file 1 — Supplementary material [file 41598_2017_10730_MOESM1_ESM.pdf]

Supplementary Information to:

Transcriptomics analysis of salt stress tolerance in the roots of the mangrove *Avicennia officinalis*

Pannaga Krishnamurthy[1, 2], Bijayalaxmi Mohanty[3], Edward Wijaya[4], Dong-Yup Lee[3,5], Tit-Meng Lim[1], Qingsong Lin[1], Jian Xu[1,6], Chiang-Shiong Loh [1, 2] and Prakash P Kumar[1, 2]*

[1] Department of Biological Sciences, National University of Singapore, 14 Science Drive 4, Singapore 117543

[2] NUS Environmental Research Institute (NERI), National University of Singapore, #02-01, T-Lab Building, 5A Engineering Drive 1, Singapore 117411

[3] Department of Chemical and Bimolecular Engineering, National University of Singapore, 4 Engineering Drive 4, Singapore 117585

[4] Department of Genome Informatics, Research Institute for Microbial Diseases, Osaka University, Osaka 565-0781, Japan

[5] Bioprocessing Technology Institute, Agency for Science, Technology and Research (A*STAR), 20 Biopolis Way, #06-01, Centros, Singapore 138668

[6] Centre for BioImaging Sciences, National University of Singapore, 14 Science Drive 4, Singapore 117557

***** Corresponding author

Address: Department of Biological Sciences, National University of Singapore, 14 Science Drive 4, Singapore 117543

Tel: +65-65162859

Fax: +65-67792486

email: dbskumar@nus.edu.sg

Supplemental Fig. S1

Length distribution of contigs and unigenes in *A.offiicnalis* root transcriptome. Transcriptome analysis was carried out from the roots of control and treated (500 mM NaCl for 24 h) seedling roots. Length distribution of contigs in control (a) and treated (b) transcriptomes with Nr annotations. Length distribution of unigenes in control (c) and treated (d) samples with Nr annotations. (e) All-unigene percentage annotations obtained with each source species using Nr database.

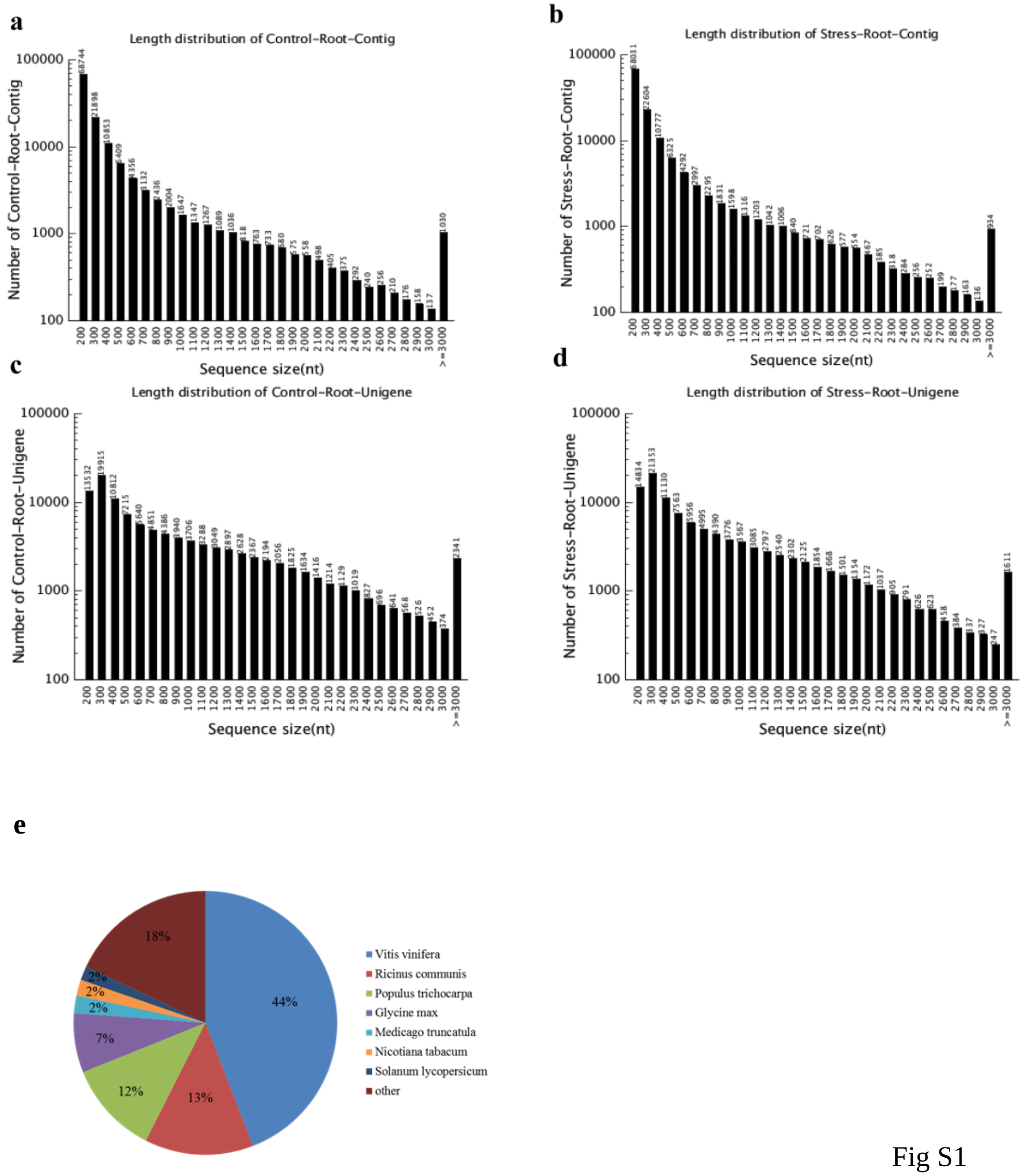

Fig S1

Supplemental Fig. S2

GO and COG classification of *A.officinalis* transcriptome: (a) Go annotations for all-unigenes were obtained using Blast2GO program. 52,746 all-unigenes were grouped into three main GO categories; biological process, cellular component and molecular function as well as into 55 sub-categories. (b) COG classification obtained from all-unigenes: 27,436 unigenes showed homology to those in COG database and were functionally classified into 25 functional classes. Number of unigenes in a specific COG cluster are shown by Y-axis.

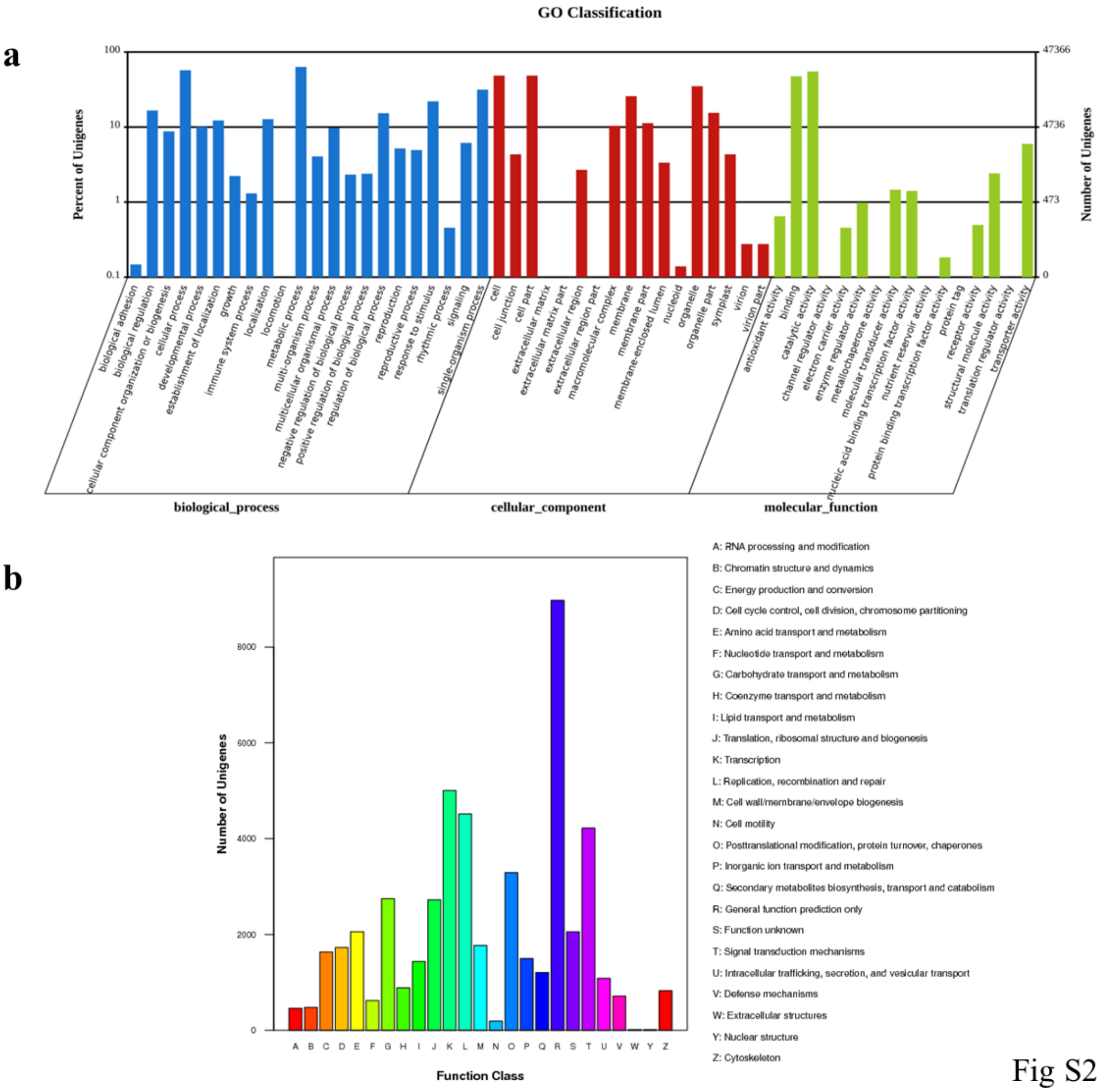

Fig S2

Supplemental Fig. S3

Expression pattern validation of selected DEGs by qRT-PCR. Changes in expression profiles of selected genes as analyzed by qRT-PCR. (a) Up-regulated and (b) down-regulated unigenes with transcript abundance in treated roots (500 mM NaCl for 24h) ≥ 2-fold compared to un-treated roots. (c) Variation in expression pattern from transcriptome data. Black bar indicates transcript abundance changes calculated by RPKM method. The grey bars plotted with error bars represent the relative expression levels quantified by qRT-PCR method. Relative expression levels of transcripts with reference to *Ubiquitin 10* transcript levels are plotted, qRT-PCR data represent means ± SD, from 3 biological replicates. Ug, unigene.

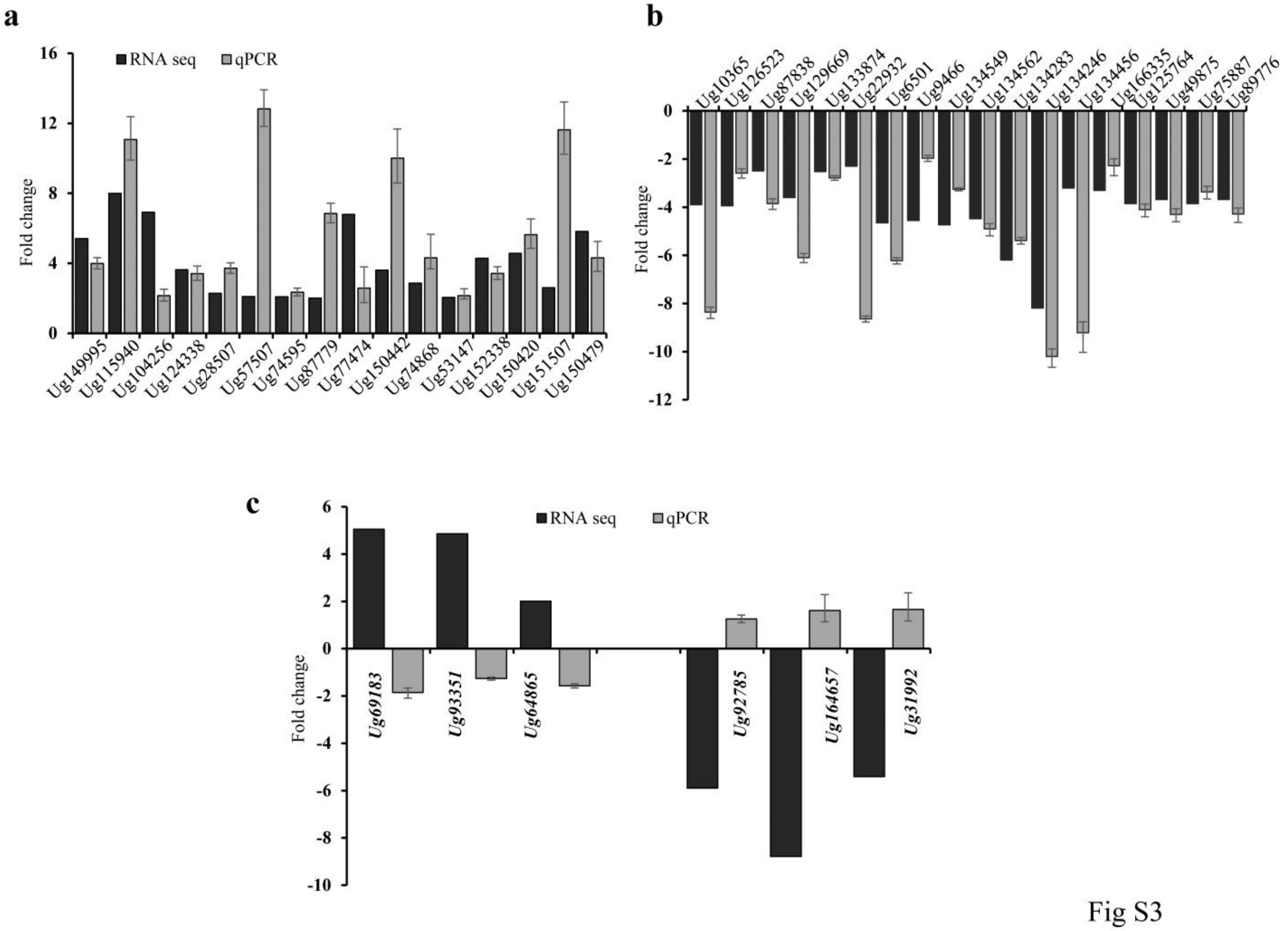

Fig S3

Supplemental Fig. S4

Temporal expression of selected DEGs: Temporal changes in the transcript levels of DEGs related to ethylene-signaling. Expression of (a) *cationic peroxidase* (b) *ethylene response factors ERF1B* and *ERF114* (c) *Hexokinase* (d) *Trihelix* transcription factor and *S-Adenosyl methionine* (*SAM2*) were monitored by qRT-PCR in *A.officinalis* roots after 500 mM NaCl treatment for varying time periods. Relative expression levels of transcripts with reference to *Ubiquitin 10* transcript levels are plotted. Data represent means ± SD from three biological replicates.

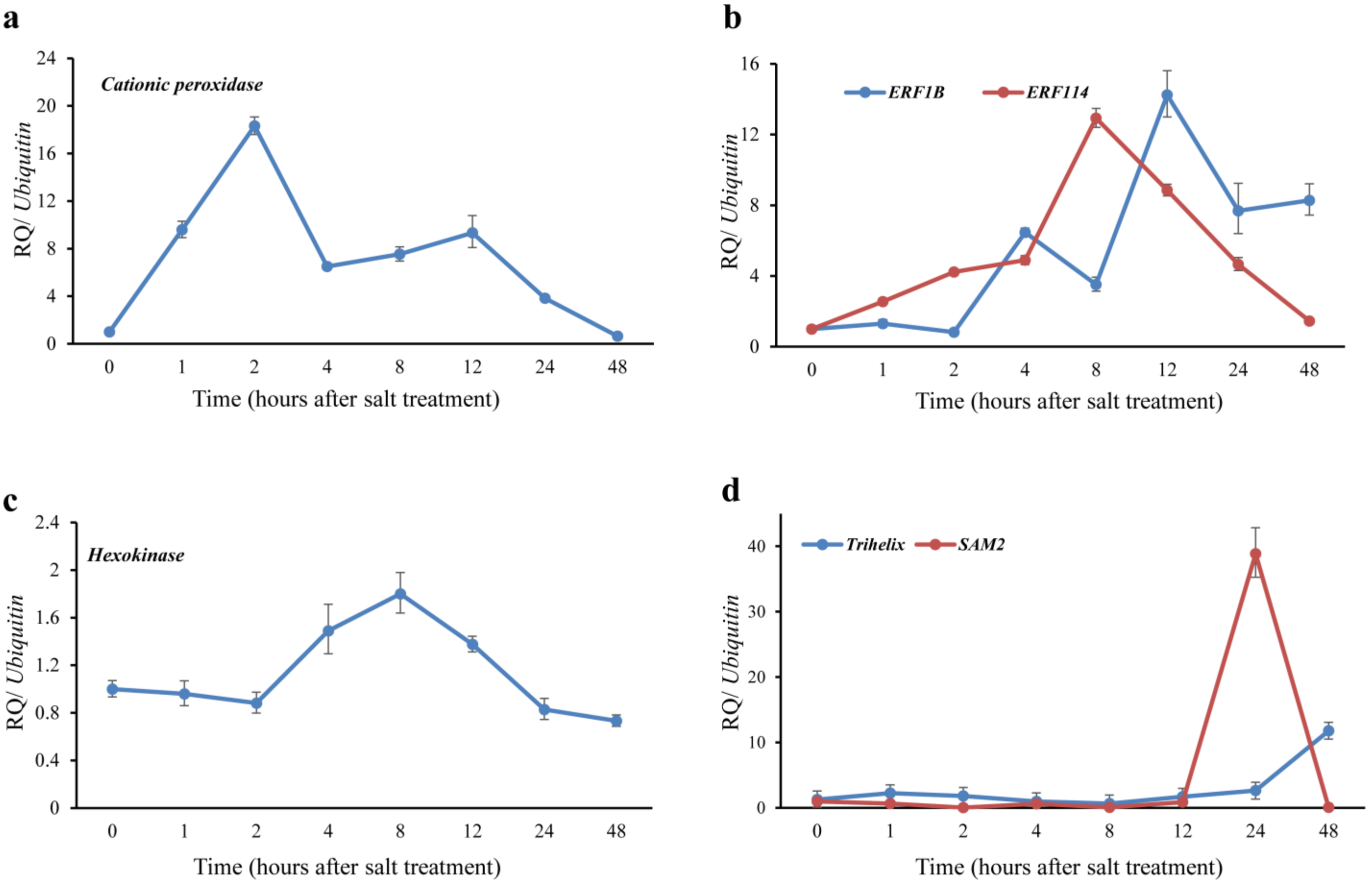

Fig S4

Supplemental Fig. S5

Temporal expression of selected DEGs: Temporal changes in the transcript levels of DEGs related to (a) auxin-signaling and (b) Ca^{2+}-signaling were monitored by qPCR in *A.officinalis* roots after 500 mM NaCl treatment for varying time periods. Relative expression levels of transcripts with reference to *Ubiquitin 10* transcript levels are plotted. Data represent means ± SD from three biological replicates. *SKOR*: *Stelar* K^{+} *outward rectifying channel*, *ARF*: *Auxin response factor*, *IAA11*: *Auxin-responsive protein11*, *PIN6*: *PIN-FORMED6*, *VHA-C*: *Vacuolar* H^{+}*-ATPase subunit c*, *VPP*: *vacuolar* H^{+}*-pyrophosphatase*, *CBL*: *calcineurin B-like protein*, *NHX*: *sodium hydrogen exchanger*, *CIPK*: *CBL-interacting serine / threonine-protein kinase*.

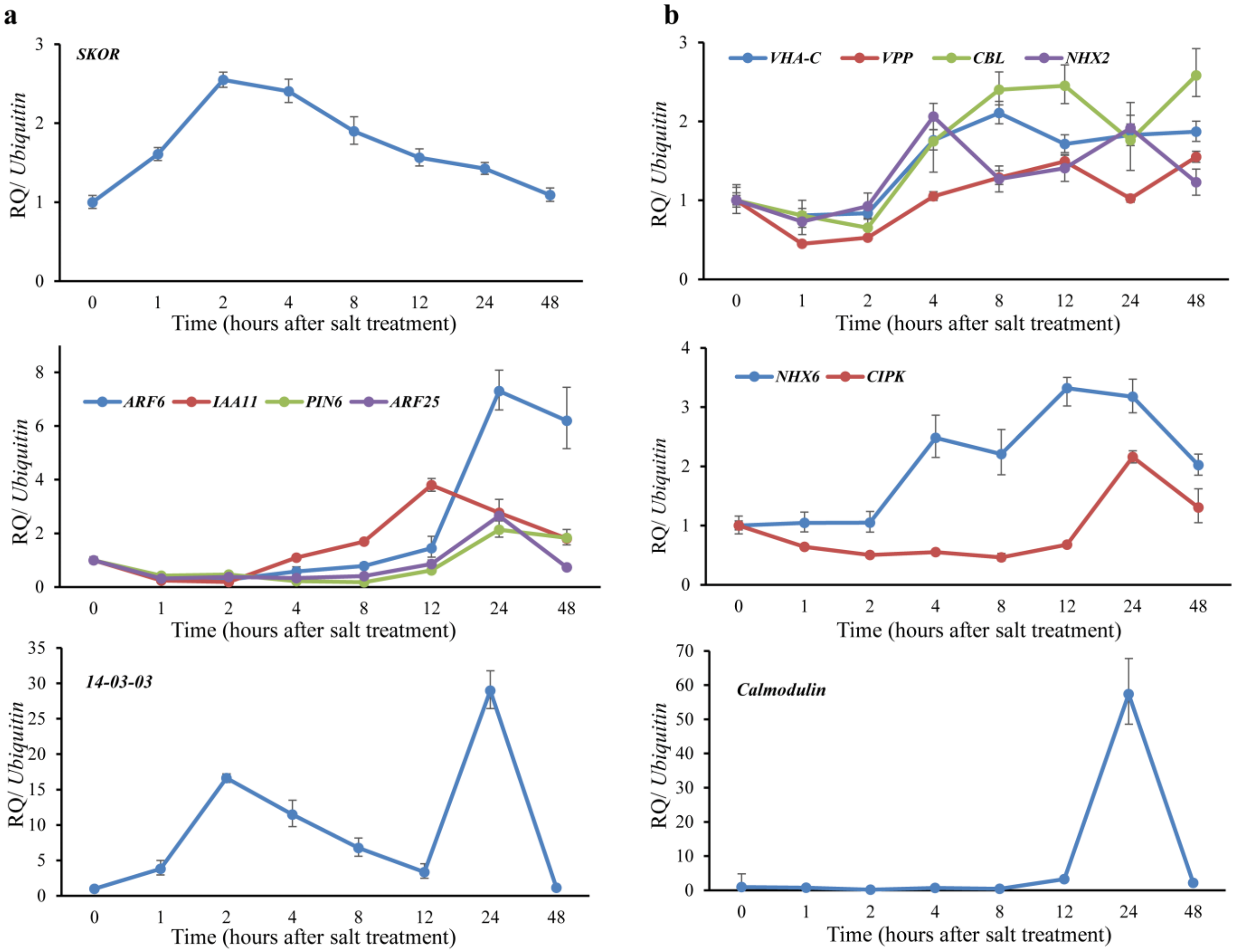

Fig S5

Supplemental Table S1

KEGG pathway analysis: In total, 42,662 unigenes were assigned to 128 KEGG pathways, while 2628 DEGs were enriched in 122 pathways.

No	Pathway	DEGs with pathway annotation (2628)	All genes with pathway annotation (42662)	Pvalue	Qvalue	Pathway ID
1	Metabolic pathways	834 (31.74%)	9390 (22.01%)	9.08e-29	3.69e-27	ko01100
2	Biosynthesis of secondary metabolites	467 (17.77%)	4578 (10.73%)	2.34e-24	7.15e-23	ko01110
3	Ribosome	213 (8.11%)	819 (1.92%)	7.56e-70	9.23e-68	ko03010
4	RNA transport	186 (7.08%)	1308 (3.07%)	6.82e-24	1.66e-22	ko03013
5	Endocytosis	134 (5.1%)	979 (2.29%)	1.41e-18	2.87e-17	ko04144
6	Spliceosome	119 (4.53%)	1517 (3.56%)	0.00342	1.13e-02	ko03040
7	Protein processing in endoplasmic reticulum	104 (3.96%)	1134 (2.66%)	0.00020	8.52e-04	ko04141
8	Glycerophospholipid metabolism	102 (3.88%)	1023 (2.4%)	9.95e-08	8.09e-07	ko00564
9	Starch and sucrose metabolism	101 (3.84%)	1126 (2.64%)	8.86e-06	4.91e-05	ko00500
10	Glycolysis / Gluconeogenesis	94 (3.58%)	584 (1.37%)	9.09e-13	1.11e-11	ko00010
11	Ether lipid metabolism	88 (3.35%)	637 (1.49%)	6.50e-14	8.81e-13	ko00565
12	mRNA surveillance pathway	87 (3.31%)	751 (1.76%)	2.01e-07	1.36e-06	ko03015
13	Citrate cycle (TCA cycle)	83 (3.16%)	290 (0.68%)	4.06e-30	2.47e-28	ko00020
14	Pentose and glucuronate interconversions	78 (2.97%)	426 (1%)	1.67e-17	2.91e-16	ko00040
15	Oxidative phosphorylation	74 (2.82%)	553 (1.3%)	1.25e-07	9.01e-07	ko00190
16	Plant-pathogen interaction	72 (2.74%)	2816 (6.6%)	1.00	1.00e+00	ko04626
17	Pyruvate metabolism	71 (2.7%)	474 (1.11%)	5.12e-10	5.67e-09	ko00620
18	Purine metabolism	66 (2.51%)	902 (2.11%)	0.03633	9.04e-02	ko00230
19	Phagosome	65 (2.47%)	436 (1.02%)	2.47e-08	2.51e-07	ko04145
20	Amino sugar and nucleotide sugar metabolism	56 (2.13%)	607 (1.42%)	0.00534	1.67e-02	ko00520
21	Plant hormone signal transduction	56 (2.13%)	2613 (6.12%)	1.00	1.00e+00	ko04075
22	Phenylpropanoid biosynthesis	53 (2.02%)	630 (1.48%)	0.02483	6.58e-02	ko00940
23	Fatty acid metabolism	51 (1.94%)	402 (0.94%)	1.04e-05	5.50e-05	ko00071
24	Proteasome	50 (1.9%)	188 (0.44%)	1.92e-16	2.92e-15	ko03050
25	Valine, leucine and isoleucine degradation	47 (1.79%)	333 (0.78%)	4.36e-06	2.80e-05	ko00280
26	Glyoxylate and dicarboxylate metabolism	46 (1.75%)	330 (0.77%)	4.99e-08	4.69e-07	ko00630
27	Alanine, aspartate and glutamate metabolism	45 (1.71%)	270 (0.63%)	8.49e-08	7.39e-07	ko00250
28	Pyrimidine metabolism	45 (1.71%)	838 (1.96%)	0.61135	9.17e-01	ko00240
29	Peroxisome	43 (1.64%)	481 (1.13%)	0.12742	2.63e-01	ko04146

30	Propanoate metabolism	42 (1.6%)	264 (0.62%)	1.24e-07	9.01e-07	ko00640
31	Ribosome biogenesis in eukaryotes	41 (1.56%)	639 (1.5%)	0.70027	9.82e-01	ko03008
32	Arginine and proline metabolism	40 (1.52%)	328 (0.77%)	0.00098	3.65e-03	ko00330
33	Cysteine and methionine metabolism	39 (1.48%)	295 (0.69%)	0.00106	3.80e-03	ko00270
34	Carbon fixation in photosynthetic organisms	38 (1.45%)	301 (0.71%)	0.00056	2.19e-03	ko00710
35	Ubiquitin mediated proteolysis	38 (1.45%)	876 (2.05%)	0.98864	1.00e+00	ko04120
36	Phenylalanine metabolism	35 (1.33%)	245 (0.57%)	8.65e-06	4.91e-05	ko00360
37	RNA degradation	33 (1.26%)	638 (1.5%)	0.92794	1.00e+00	ko03018
38	Ascorbate and aldarate metabolism	30 (1.14%)	243 (0.57%)	0.00012	5.21e-04	ko00053
39	Fructose and mannose metabolism	31 (1.18%)	277 (0.65%)	0.00303	1.03e-02	ko00051
40	Pentose phosphate pathway	31 (1.18%)	219 (0.51%)	1.58e-05	8.02e-05	ko00030
41	Galactose metabolism	31 (1.18%)	398 (0.93%)	0.07165	1.59e-01	ko00052
42	Glutathione metabolism	29 (1.1%)	242 (0.57%)	0.00096	3.65e-03	ko00480
43	beta-Alanine metabolism	28 (1.07%)	168 (0.39%)	5.96e-06	3.63e-05	ko00410
44	Tryptophan metabolism	28 (1.07%)	201 (0.47%)	2.26e-05	1.06e-04	ko00380
45	Tyrosine metabolism	28 (1.07%)	265 (0.62%)	0.00153	5.32e-03	ko00350
46	Butanoate metabolism	27 (1.03%)	228 (0.53%)	0.00043	1.73e-03	ko00650
47	alpha-Linolenic acid metabolism	27 (1.03%)	234 (0.55%)	0.06158	1.39e-01	ko00592
48	RNA polymerase	27 (1.03%)	365 (0.86%)	0.08527	1.81e-01	ko03020
49	Glycerolipid metabolism	26 (0.99%)	302 (0.71%)	0.03497	8.88e-02	ko00561
50	Biosynthesis of unsaturated fatty acids	25 (0.95%)	194 (0.45%)	0.00365	1.17e-02	ko01040
51	Glycine, serine and threonine metabolism	24 (0.91%)	242 (0.57%)	0.18582	3.48e-01	ko00260
52	Valine, leucine and isoleucine biosynthesis	22 (0.84%)	128 (0.3%)	1.70e-05	8.31e-05	ko00290
53	ABC transporters	22 (0.84%)	589 (1.38%)	0.99943	1.00e+00	ko02010
54	Limonene and pinene degradation	21 (0.8%)	172 (0.4%)	0.02085	5.78e-02	ko00903
55	Nitrogen metabolism	21 (0.8%)	206 (0.48%)	0.02837	7.36e-02	ko00910
56	Flavonoid biosynthesis	21 (0.8%)	284 (0.67%)	0.12981	2.64e-01	ko00941
57	Natural killer cell mediated cytotoxicity	20 (0.76%)	258 (0.6%)	0.48767	8.04e-01	ko04650
58	Lysine degradation	19 (0.72%)	186 (0.44%)	0.01410	4.09e-02	ko00310
59	Carotenoid biosynthesis	18 (0.68%)	231 (0.54%)	0.47815	7.99e-01	ko00906
60	Cyanoamino acid metabolism	18 (0.68%)	276 (0.65%)	0.54652	8.77e-01	ko00460
61	Terpenoid backbone biosynthesis	17 (0.65%)	395 (0.93%)	0.84134	1.00e+00	ko00900
62	Selenocompound metabolism	16 (0.61%)	71 (0.17%)	9.16e-05	4.14e-04	ko00450
63	Cutin, suberine and wax biosynthesis	15 (0.57%)	156 (0.37%)	0.05048	1.18e-01	ko00073
64	Phenylalanine, tyrosine and tryptophan biosynthesis	15 (0.57%)	184 (0.43%)	0.16164	3.08e-01	ko00400
65	Aminoacyl-tRNA biosynthesis	15 (0.57%)	258 (0.6%)	0.52380	8.52e-01	ko00970
66	Phosphatidylinositol signaling system	15 (0.57%)	496 (1.16%)	0.99844	1.00e+00	ko04070
67	Fatty acid biosynthesis	14 (0.53%)	121 (0.28%)	0.02029	5.76e-02	ko00061
68	Steroid biosynthesis	13 (0.49%)	180 (0.42%)	0.47051	7.97e-01	ko00100
69	Basal transcription factors	13 (0.49%)	265 (0.62%)	0.76221	1.00e+00	ko03022
70	Diterpenoid biosynthesis	13 (0.49%)	115 (0.27%)	0.00746	2.27e-02	ko00904
71	Inositol phosphate metabolism	13 (0.49%)	373 (0.87%)	0.97890	1.00e+00	ko00562
72	Sulfur metabolism	12 (0.46%)	111 (0.26%)	0.04168	1.02e-01	ko00920
73	Protein export	12 (0.46%)	195 (0.46%)	0.58637	9.07e-01	ko03060

74	Sphingolipid metabolism	12 (0.46%)	231 (0.54%)	0.79248	1.00e+00	ko00600
75	Nucleotide excision repair	12 (0.46%)	329 (0.77%)	0.97530	1.00e+00	ko03420
76	Base excision repair	11 (0.42%)	224 (0.53%)	0.83168	1.00e+00	ko03410
77	Histidine metabolism	11 (0.42%)	82 (0.19%)	0.05840	1.34e-01	ko00340
78	Pantothenate and CoA biosynthesis	11 (0.42%)	138 (0.32%)	0.13445	2.69e-01	ko00770
79	Ubiquinone and other terpenoid-quinone biosynthesis	11 (0.42%)	174 (0.41%)	0.31299	5.57e-01	ko00130
80	Isoquinoline alkaloid biosynthesis	10 (0.38%)	85 (0.2%)	0.08397	1.81e-01	ko00950
81	SNARE interactions in vesicular transport	10 (0.38%)	209 (0.49%)	0.61739	9.17e-01	ko04130
82	Zeatin biosynthesis	10 (0.38%)	210 (0.49%)	0.58760	9.07e-01	ko00908
83	Regulation of autophagy	10 (0.38%)	278 (0.65%)	0.97016	1.00e+00	ko04140
84	Stilbenoid, diarylheptanoid and gingerol biosynthesis	9 (0.34%)	228 (0.53%)	0.89149	1.00e+00	ko00945
85	Vitamin B6 metabolism	8 (0.3%)	56 (0.13%)	0.01208	3.59e-02	ko00750
86	Circadian rhythm - mammal	8 (0.3%)	86 (0.2%)	0.14806	2.87e-01	ko04710
87	Synthesis and degradation of ketone bodies	8 (0.3%)	132 (0.31%)	0.30754	5.57e-01	ko00072
88	Porphyrin and chlorophyll metabolism	8 (0.3%)	208 (0.49%)	0.90906	1.00e+00	ko00860
89	Circadian rhythm - plant	8 (0.3%)	431 (1.01%)	0.99999	1.00e+00	ko04712
90	Nicotinate and nicotinamide metabolism	7 (0.27%)	60 (0.14%)	0.08615	1.81e-01	ko00760
91	Tropane, piperidine and pyridine alkaloid biosynthesis	7 (0.27%)	64 (0.15%)	0.25558	4.72e-01	ko00960
92	Fatty acid elongation	7 (0.27%)	100 (0.23%)	0.56070	8.88e-01	ko00062
93	Other glycan degradation	7 (0.27%)	214 (0.5%)	0.98897	1.00e+00	ko00511
94	Glycosphingolipid biosynthesis - globo series	6 (0.23%)	44 (0.1%)	0.04998	1.18e-01	ko00603
95	Linoleic acid metabolism	6 (0.23%)	73 (0.17%)	0.87559	1.00e+00	ko00591
96	One carbon pool by folate	6 (0.23%)	84 (0.2%)	0.35782	6.15e-01	ko00670
97	Homologous recombination	6 (0.23%)	273 (0.64%)	0.99419	1.00e+00	ko03440
98	N-Glycan biosynthesis	5 (0.19%)	288 (0.68%)	0.99970	1.00e+00	ko00510
99	Taurine and hypotaurine metabolism	4 (0.15%)	37 (0.09%)	0.31494	5.57e-01	ko00430
100	Riboflavin metabolism	4 (0.15%)	60 (0.14%)	0.63125	9.17e-01	ko00740
101	Flavone and flavonol biosynthesis	4 (0.15%)	94 (0.22%)	0.68589	9.80e-01	ko00944
102	Glycosaminoglycan degradation	4 (0.15%)	130 (0.3%)	0.96423	1.00e+00	ko00531
103	Biotin metabolism	3 (0.11%)	9 (0.02%)	0.02434	6.58e-02	ko00780
104	Arachidonic acid metabolism	3 (0.11%)	47 (0.11%)	0.62976	9.17e-01	ko00590
105	C5-Branched dibasic acid metabolism	3 (0.11%)	19 (0.04%)	0.14239	2.80e-01	ko00660
106	Brassinosteroid biosynthesis	3 (0.11%)	121 (0.28%)	0.87969	1.00e+00	ko00905
107	Sulfur relay system	3 (0.11%)	45 (0.11%)	0.60709	9.17e-01	ko04122
108	Lysine biosynthesis	3 (0.11%)	81 (0.19%)	0.99391	1.00e+00	ko00300
109	Mismatch repair	3 (0.11%)	173 (0.41%)	0.99973	1.00e+00	ko03430
110	DNA replication	3 (0.11%)	228 (0.53%)	0.99993	1.00e+00	ko03030
111	Glycosylphosphatidylinositol(GPI)-anchor biosynthesis	3 (0.11%)	238 (0.56%)	0.99994	1.00e+00	ko00563
112	Benzoxazinoid biosynthesis	2 (0.08%)	52 (0.12%)	0.69136	9.81e-01	ko00402
113	Glucosinolate biosynthesis	2 (0.08%)	52 (0.12%)	0.74246	1.00e+00	ko00966
114	Glycosphingolipid biosynthesis - ganglio series	2 (0.08%)	83 (0.19%)	0.97101	1.00e+00	ko00604

115	Photosynthesis	2 (0.08%)	118 (0.28%)	0.99678	1.00e+00	ko00195
116	Anthocyanin biosynthesis	1 (0.04%)	11 (0.03%)	0.35135	6.12e-01	ko00942
117	Photosynthesis - antenna proteins	1 (0.04%)	29 (0.07%)	0.84121	1.00e+00	ko00196
118	Folate biosynthesis	1 (0.04%)	41 (0.1%)	0.91708	1.00e+00	ko00790
119	Thiamine metabolism	1 (0.04%)	46 (0.11%)	0.92823	1.00e+00	ko00730
120	Isoflavonoid biosynthesis	1 (0.04%)	51 (0.12%)	0.93078	1.00e+00	ko00943
121	Sesquiterpenoid and triterpenoid biosynthesis	1 (0.04%)	61 (0.14%)	0.98180	1.00e+00	ko00909
122	Non-homologous end-joining	1 (0.04%)	65 (0.15%)	0.98535	1.00e+00	ko03450
123	Other types of O-glycan biosynthesis	--	55 (0.13%)	--	--	ko00514
124	Indole alkaloid biosynthesis	--	24 (0.06%)	--	--	ko00901
125	Monoterpenoid biosynthesis	--	21 (0.05%)	--	--	ko00902
126	Lipoic acid metabolism	--	18 (0.04%)	--	--	ko00785
127	Betalain biosynthesis	--	6 (0.01%)	--	--	ko00965
128	Caffeine metabolism	--	4 (0.01%)	--	--	ko00232

Table S2: List of differentially expressed transporters/channels

GeneID	Control_Root_1, Control_Root_2 RPKM	Stress_Root_1,Stress_Root_2 RPKM	log2Ratio(Stress_Root_1,Stress_Root_2/Control_Root_1,Control_Root_2) normalize	p-value	Homologous species	Swissprot-annotation	Up-Down-Regulation(Stress_Root_1,Stress_Root_2/Control_Root_1,Control_Root_2)
**Ion transporters**							
Unigene152563	1.2855	5.8442	2.008697229	3.35453E-05	*P. trichocarpa*	Phosphate transporter PHO1 homolog 1	up
Unigene107038	0.00845	0.04135	2.290864083	0.04773992	*P. trichocarpa*	Potassium channel SKOR	up
Unigene82608	0.001	0.09615	6.587214952	0.01417369	*V. vinifera*	Potassium transporter 13	up
Unigene251	0	0.3797	5.219264215	9.3751E-06	*R. communis*	Probable cyclic nucleotide-gated ion channel 20	up
Unigene35000	0	0.5712	5.371267308	2.84275E-06	*V. vinifera*	Probable peptide/nitrate transporter At2g40460	up
Unigene150472	0.1812	5.4618	4.907320209	2.9248E-15	*C. subellipsoidea*	sodium-dependent inorganic phosphate cotransporter	up
Unigene164554	0.001	0.12375	6.951284715	0.01413374	*V. vinifera*	Cation/calcium exchanger 3 OS=Arabidopsis thaliana	up
Unigene91704	0.001	0.04705	5.556122818	0.0147219	*V. vinifera*	Vacuolar cation/proton exchanger 2	up
Unigene84902	0.0694	0.6033	3.219264215	0.000110988	*R. communis*	Magnesium transporter	up
Unigene73148	0.1473	0.7133	2.164816431	0.000176754	*V. vinifera*	Molybdate transporter 1 MOT1	up
Unigene30853	0.0614	1.0809	4.049339214	7.19668E-05	*P. trichocarpa*	Nitrate transporter	up
Unigene16087	0.1068	0.9455	2.956229809	1.61896E-06	*S. tuberosum*	bile acid:Na+ symporter, BASS family	up
Unigene25620	1.2064	5.1426	2.148874887	6.08046E-05	*A. thaliana*	Putative peptide/nitrate transporter At2g38100	up

Unigene51908	1.9328	4.9367	1.352854616	0.03710494	*P. maritima*	Sodium/hydrogen exchanger 2 NHX2	up
Unigene115806	1.26635	3.21865	1.345779506	0.02027964	*A. lyrata*	Sodium/hydrogen exchanger 6NHX6	up
Unigene18584	2.1834	0.2253	-3.120585788	0.000179145	*N. tabacum*	Potassium channel KAT3 KAT3	down
Unigene18716	0.4676	0	-4.758015709	0.000148525	*V. vinifera*	Potassium transporter 23 HAK23	down
Unigene137762	4.8067	0.42	-3.535623287	5.07619E-06	*G. max*	Probable anion transporter 5 PHT4;5	down
Unigene62793	1.6122	0	-5.038123628	2.47977E-05	*N. tabacum*	Probable potassium channel AKT5	down
Unigene130662	25.1943	1.0099	-4.633234084	4.0321E-144	*C. variabilis*	Probable voltage-gated potassium channel subunit beta	down
Unigene130663	22.6262	0.8896	-4.662735205	1.634E-112	*B. distachyon*	Probable voltage-gated potassium channel subunit beta	down
Unigene135654	3.512	0	-5.1985883	7.69084E-06	*S. moellendorffii*	Uncharacterized protein involved in cation transport	down
Unigene134767	5.8469	0.5741	-3.342978209	7.24766E-17	*P. sojae*	Vacuolar cation/proton exchanger 1a CAX1a	down
Unigene133281	3.2554	0.3533	-3.1985883	1.55914E-11	*T. oceanica*	Vacuolar cation/proton exchanger 2 CAX2	down
Unigene126523	2.8779	0.5649	-2.32917241	4.98453E-08	*V. vinifera*	Vacuolar cation/proton exchanger 3 CAX3	down
Unigene134901	5.5253	0.7121	-2.950660786	2.55146E-09	*P. infestans*	Vacuolar cation/proton exchanger 3 CAX3	down
Unigene134902	8.5135	0.4083	-4.376925541	2.47523E-20	*Z. mays*	Vacuolar cation/proton exchanger 4 CAX4	down
Unigene138829	3.0972	0	-4.857551382	8.13932E-05	*M. truncatula*	Vacuolar iron transporter 1 VIT1	down
Unigene135731	3.0512	0	-5.272588881	4.30982E-06	--	Ca2+/H+ antiporter	down
Unigene134016	3.5373	0	-5.950660786	5.42407E-09	--	Ca2+/H+ antiporter	down
Unigene69615	0.2514	0.10135	-1.310638561	0.04087325	*R. communis*	Cation-chloride cotransporter 1CCC1	down
Unigene27252	0.10255	0.001	-6.680183682	0.04862699	*N. tabacum*	Cyclic nucleotide-gated ion channel 1 CNGC1	down
Unigene130311	2.2038	0	-4.758015709	0.000148525	*V. carteri*	Mg2+ and Co2+ transporters	down

Unigene102238	75.6421	4.6622	-4.014402917	0	*A. thaliana*	Probable peptide/nitrate transporter At1g72130	down
Unigene134419	5.7008	0	-7.079943803	3.14887E-17	*M. pusilla*	Probable peptide/nitrate transporter At3g43790	down
Unigene131825	5.6123	0	-5.950660786	5.42407E-09	--	High-affinity Fe2+/Pb2+ permease	down
Unigene134030	3.3815	0.1516	-4.474222743	1.56486E-06	*B. hominis*	Metal tolerance protein 12 MTP12	down
Unigene17669	0.4322	0.063	-2.808641782	6.24054E-06	*P. trichocarpa*	Metal tolerance protein C2	down
Unigene62963	25.533	2.626	-3.275414925	1.70947E-92	*A. laibachii*	Mitochondrial outer membrane protein porin 2 VDAC2	down
Unigene75605	6.8156	1.2204	-2.522912235	3.83365E-17	*G. max*	Nitrate transporter 1.2 NRT1.2	down
Unigene124132	3.2163	0.588	-2.410092405	2.84281E-20	*P. trichocarpa*	Sulfate transporter 3.1 SULTR3;1	down
Unigene135158	3.408	0.1673	-4.342978209	1.15863E-10	*Oryza sativa*	HKT1	down
**ATPases**							
Unigene35552	0.8624	4.5383	2.551839554	1.46283E-28	V. vinifera	Calcium-transporting ATPase 12, plasma membrane-type ACA12	up
Unigene115940	0.001	0.25455	7.991805255	0.006822052	M. truncatula	Calcium-transporting ATPase 2, plasma membrane-type ACA2	up
Unigene150699	0.3569	5.8733	3.900195774	6.3372E-23	R. fluitans	Calcium-transporting ATPase 4, endoplasmic reticulum-type ECA4	up
Unigene155730	0.001	0.5544	9.114783447	0.04636662	A. thaliana	V-type proton ATPase subunit B2 VHA-B2	up
Unigene3470	0.001	0.12775	6.997179481	0.01416891	V. vinifera	V-type proton ATPase subunit C VHA-C	up
Unigene88787	4.2021	27.5853	2.648376899	6.31043E-48	D. dichotoma	ATP synthase subunit 9, mitochondrial ATP9	up
Unigene152275	0.8501	4.9601	2.312373619	1.12483E-07	Z. mays	ATP synthase subunit alpha, mitochondrial ATPA	up
Unigene150523	1.6718	8.9181	2.271731635	6.38215E-14	H. vulgare	ATP synthase subunit beta-3	up
Unigene152794	0.1392	3.2063	4.219264215	2.04539E-05	Z. mays	ATP synthase subunit O	up
Unigene29379	0.49575	1.23145	1.312673374	0.04816	V. radiata	Pyrophosphate-energized vacuolar membrane proton pump	up

Unigene152156	0.3294	4.794	3.856694136	2.32401E-07	G. theta	Soluble inorganic pyrophosphatase 1, PPA1	up
Unigene134387	4.17	0.47	-3.152294648	1.77E-09	*V. vinifera*	Phospholipid-transporting ATPase 10, ALA10	down
Unigene132087	3.93	0.16	-4.651100505	2.63E-07	*E. siliculosus*	Phospholipid-transporting ATPase 2, ALA2	down
Unigene134685	3.11	0.24	-3.705548289	8.49E-07	*O. sativa*	Phospholipid-transporting ATPase 6, ALA6	down
Unigene87855	41.12	3.12	-3.716097759	0	*S. lycopersicum*	Plasma membrane ATPase 1, PMA1	down
Unigene130898	10.86	1.10	-3.29519031	3.13E-36	*O. sativa*	Probable calcium-transporting ATPase 5, plasma membrane-type	down
Unigene132768	2.42	0.34	-2.825129904	7.91E-05	*A. thaliana*	Probable cation-transporting ATPase	down
Unigene135086	5.21	0.10	-5.705548289	8.07E-27	*P. sojae*	Probable inorganic phosphate transporter 1-5, PHT1-5	down
Unigene135087	12.40	1.08	-3.515445405	6.98E-42	*A. lyrata*	Probable inorganic phosphate transporter 1-6 , PHT1-6	down
Unigene136395	2.48	0.00	-4.758015709	0.000148525	*O. sativa*	Probable inorganic phosphate transporter 1-8 , PHT1-8	down
Unigene131458	2.64	0.13	-4.342978209	5.19E-06	*S. bicolor*	Probable V-type proton ATPase subunit d	down
Unigene133821	4.36	0.00	-5.808641782	2.76E-08	*V. carteri*	Probable V-type proton ATPase subunit H	down
Unigene11168	33.41	2.62	-3.667651681	6.03E-155	*M.truncatula*	Soluble inorganic pyrophosphatase 1	down
Unigene133714	3.91	0.00	-5.950660786	5.42E-09	*P. patens*	Vacuolar H+-ATPase V1 sector, subunit C	down
Unigene131990	2.55	0.00	-5.120585788	1.38E-05	--	Vacuolar H+-ATPase V1 sector, subunit H	down
Unigene131766	6.34	0.33	-4.272588881	3.77E-10	--	vacuolar-type H+-ATPase subunit E	down
Unigene133043	3.35	0.17	-4.272588881	9.47E-06	--	V-type H+-transporting ATPase subunit E	down

Unigene134998	8.26	1.21	-2.768822463	5.39E-22	*L. japonicus*	V-type proton ATPase 16 kDa proteolipid subunit, VATP-P1	down
Unigene132841	3.34	0.19	-4.120585788	3.18E-05	*T. oceanica*	V-type proton ATPase catalytic subunit A (Fragment)	down
Unigene134162	6.59	0.18	-5.1985883	2.51E-10	*C. subellipsoidea*	V-type proton ATPase catalytic subunit A isoform 1	down
Unigene132840	5.38	0.36	-3.881398124	5.51E-14	*H. vulgare*	V-type proton ATPase catalytic subunit A	down
Unigene129698	4.51	0.13	-5.140485345	1.98E-18	*G. hirsutum*	V-type proton ATPase subunit B 1	down
Unigene126678	12.24	2.33	-2.1985883	2.26E-19	*G. hirsutum*	V-type proton ATPase subunit B 2 (Fragment)	down
Unigene130688	3.21	0.00	-5.535623287	4.41E-07	*V. carteri*	V-type proton ATPase subunit B 2	down
Unigene22183	2.47	0.17	-3.687626381	2.20E-09	*M. crystallinum*	V-type proton ATPase subunit B2, VHA-B2	down
Unigene136061	2.80	0.00	-4.857551382	8.14E-05	*C. subellipsoidea*	V-type proton ATPase subunit D, VHA-D	down
Unigene132829	3.96	0.21	-4.1985883	1.73E-05	*P. patens*	V-type proton ATPase subunit F, VHA-F	down
Unigene134952	10.04	0.97	-3.360051723	4.09E-17	*H. vulgare*	V-type proton ATPase subunit G1, VHA-G1	down
Unigene138460	17.97	2.34	-2.950660786	2.94E-13	*Chondrus crispus*	ATP synthase subunit 9, mitochondrial, ATP9	down
Unigene100890	29.43	2.36	-3.637013867	1.40E-237	*H. vulgare*	ATP synthase subunit alpha, mitochondrial, ATPA	down
Unigene23298	37.27	3.40	-3.447805408	4.88E-267	*H. vulgare*	ATP synthase subunit beta-1, mitochondrial	down
Unigene76853	14.85	1.39	-3.410092405	4.41E-32	*O. lucimarinus*	ATP synthase subunit delta', mitochondrial	down
Unigene135201	10.48	0.66	-3.976195879	1.35E-48	*H. vulgare*	ATP synthase subunit gamma, mitochondrial, ATPC	down
Unigene131726	6.97	0.47	-3.881398124	2.07E-26	*M. pusilla*	Inorganic pyrophosphatase/exopolyphosphatase	down

Unigene135562	4.23	0.11	-5.272588881	3.87E-20	*P. patens*	Cation-transporting ATPase CA1	down
Unigene135689	4.82	0.15	-4.995054906	4.40E-09	*S.moellendorffii*	Copper-transporting ATPase RAN1	down
Unigene144754	0.97	0.13	-3.120585788	0.000179145	*M. truncatula*	Calcium-transporting ATPase 1, ACA1	down
Unigene135366	10.66	1.56	-2.768284044	4.69E-23	*P. sojae*	Calcium-transporting ATPase 12, plasma membrane-type, ACA12	down
Unigene135582	4.70	0.00	-7.325700218	7.95E-20	*R. fluitans*	Calcium-transporting ATPase 4, ECA4	down
Unigene131018	6.14	0.52	-3.555522845	2.24E-43	*P. trichocarpa*	Calcium-transporting ATPase 4, plasma membrane-type, ACA4	down
Unigene132961	8.49	0.31	-4.783550801	2.35E-27	*M. polymorpha*	Cation transport ATPase	down
**Sugar and osmolyte transporters**							
Unigene13936	0.001	0.0646	6.01346226	0.01434315	*S. demissum*	Sucrose transport protein SUC3	up
Unigene6107	0.0659	0.3908	2.271731635	6.09989E-05	*O. europaea*	Sugar carrier protein C, STC	up
Unigene152267	0.3726	4.9925	3.634301714	9.31565E-09	*H. vulgare*	Sugar transport protein 11, STP11	up
Unigene150777	0	8.2926	7.258792579	1.70599E-18	*H. vulgare*	Sugar transport protein 8, STP8	up
Unigene150178	0.6485	7.2812	3.482298621	4.5454E-12	*H. vulgare*	Sugar transport protein 9, STP9	up
Unigene129526	1.4483	6.608	2.18319496	4.98346E-11	*M. truncatula*	Probable inositol transporter 2, INT2	up
Unigene78096	0	0.4664	5.049339214	3.14848E-05	*V. vinifera*	Probable sugar phosphate/phosphate translocator	up
Unigene124050	0.0534	0.4956	3.371267308	3.24208E-05	*V. vinifera*	Probable plastidic glucose transporter 2	up
Unigene151004	1.1596	5.1247	2.049339214	0.000170902	*M. pusilla*	Probable sugar phosphate/phosphate translocator	up
Unigene68405	0.001	0.09515	6.572131751	0.01422354	*R. communis*	UDP-galactose transporter 1, UDP-GALT1	up
Unigene22561	0.2286	0.9248	2.164816431	0.000176754	*V. vinifera*	Bidirectional sugar transporter SWEET1b	up
Unigene133074	7.2245	0	-7.520516395	3.75782E-22	*P. patens*	Fucose permease	down
Unigene135688	3.2887	0	-5.410092405	1.36931E-06	*P. patens*	Fucose permease	down

Unigene133355	1.9862	0	-4.758015709	0.000148525	*P. infestans*	Glycerol uptake facilitator and related permeases	down
Unigene133988	4.3775	0	-6.038123628	1.85809E-09	*H. vulgare*	Inositol transporter 4, INT4	down
Unigene138409	5.78	1.1093	-2.209395055	2.05617E-09	*V. vinifera*	Monosaccharide-sensing protein 2, MSSP2	down
Unigene130336	14.1833	0.2575	-5.778479811	1.6507E-133	*P. trichocarpa*	Probable inositol transporter 2, INT2	down
Unigene135113	6.1353	0.275	-4.474222743	6.65305E-27	*S. bicolor*	Probable polyol transporter 3, PLT3	down
Unigene128188	9.1846	0.7777	-3.556684903	4.17683E-41	*M. domestica*	Probable polyol transporter 6, PLT6	down
Unigene135345	16.1209	0.7203	-4.479039759	1.98049E-67	*M. truncatula*	Probable sugar phosphate/phosphate translocator	down
Unigene135346	13.0094	0.4065	-4.995054906	2.46731E-39	*A. thaliana*	Probable sugar phosphate/phosphate translocator	down
Unigene133794	4.3769	0.1003	-5.442513883	4.86458E-12	*E. siliculosus*	GDP-fucose transporter	down
Unigene114741	20.5643	1.9082	-3.424591975	4.3399E-149	*P. sitchensis*	Sugar transport protein 10, STP10	down
Unigene127030	29.2446	2.2135	-3.717373724	8.2771E-194	*H. vulgare*	Sugar transport protein 11, STP11	down
Unigene89374	28.7055	2.3825	-3.585512163	4.4412E-215	*P. sitchensis*	Sugar transport protein 13, STP13	down
Unigene135097	12.9101	1.193	-3.426394217	1.32622E-34	*A. lyrata*	Sugar transport protein 2, STP2	down
Unigene63988	23.9023	2.4745	-3.26668828	1.8481E-174	*A. lyrata*	Sugar transport protein 4, STP4	down
Unigene10717	28.6709	1.8588	-3.941908312	9.4804E-203	*P. sitchensis*	Sugar transport protein 7, STP7	down
Unigene135774	3.5098	0.6959	-2.32917241	0.000115707	*P. sojae*	Sugar transport protein 9, STP9	down
Unigene52003	11.5679	1.0895	-3.403172991	4.54961E-78	*G. max*	H(+)/hexose cotransporter 3, HUP3	down
Unigene123418	37.2275	5.7531	-2.6049025	6.1197E-204	*R. communis*	Hexose carrier protein HEX6	down
Unigene32454	0.7045	0.0336	-4.120585788	3.17901E-05	*R. communis*	Probable polyamine transporter	down
**Carriers and permeases**							
Unigene151569	0.7235	7.3839	3.153675873	3.23352E-09	*T. oceanica*	Nucleoside permease	up
Unigene150480	1.9086	13.3117	2.578592282	1.2955E-38	*C. subellipsoidea*	Permeases of the major facilitator superfamily	up
Unigene150929	0.2168	5.9737	4.777259668	5.75269E-14	*C. variabilis*	Permeases of the major facilitator superfamily	up

Unigene152024	0.27	5.3332	4.297266727	4.99539E-10	--	Permeases of the major facilitator superfamily	up
Unigene151505	0	5.9173	5.956229809	9.15877E-09	--	Permeases of the major facilitator superfamily	up
Unigene151730	0	6.5601	5.956229809	9.15877E-09	--	Permeases of the major facilitator superfamily	up
Unigene151451	0.3927	6.1242	3.956229809	6.77214E-08	--	Permeases of the major facilitator superfamily	up
Unigene152986	0	2.8686	5.136802055	1.71399E-05	*P. infestans*	Permeases of the major facilitator superfamily	up
Unigene150413	0.5673	4.9145	3.108232903	7.34146E-06	*Z. mays*	Permeases of the major facilitator superfamily	up
Unigene40195	0.11745	0.2396	1.028581195	0.01220935	*A. thaliana*	Permeases of the major facilitator superfamily	up
Unigene152580 l	0.5635	6.1652	3.464376713	1.06429E-07	*H. vulgare*	Adenine/guanine permease AZG2	up
Unigene125771	19.012	83.8693	2.059755829	6.3971E-217	*Z. mays*	ADP,ATP carrier protein 1, mitochondrial, ANT1	up
Unigene37930	27.0312	158.5641	2.501327849	3.8832E-164	*H. vulgare*	ADP,ATP carrier protein, mitochondrial	up
Unigene140581	0.2242	1.7131	2.923808331	4.41035E-05	*R. communis*	Solute carrier	up
Unigene149797	0	2.9664	5.508770832	8.76527E-07	*E. siliculosus*	Thylakoid ADP,ATP carrier protein, chloroplastic, TAAC	up
Unigene12434	12.8988	1.9195	-2.743218707	3.27926E-16	*Micromonas sp.*	Acyl carrier protein 2, mitochondrial, MTACP2	down
Unigene130941	4.127	0.876	-2.230768706	1.74865E-05	*P. trichocarpa*	Adenine/guanine permease AZG1	down
Unigene62185	38.9368	2.9268	-3.659341135	8.3831E-240	*R. communis*	Adenine/guanine permease AZG2	down
Unigene126453	32.5577	3.9667	-3.073456701	2.8066E-178	*V. vinifera*	ADP,ATP carrier protein 3, AAC3	down
Unigene119139	12.0904	1.9572	-2.428708083	5.22802E-17	*G. max*	solute carrier family 25 (mitochondrial oxoglutarate transporter), member 11	down
Unigene139293	9.8717	0.8611	-3.535623287	5.49152E-13	*V. vinifera*	solute carrier family 25 (mitochondrial oxoglutarate transporter), member 11	down

Unigene134093	4.2774	0	-6.308212791	4.72315E-11	*K. micrum*	Mitochondrial carnitine/acylcarnitine carrier-like protein	down
Unigene132160	3.3872	0.2117	-3.995054906	2.38452E-08	*P. sojae*	Mitochondrial import inner membrane translocase subunit TIM50	down
Unigene134445	3.4018	0.167	-4.342978209	5.18747E-06	*O. sativa*	Mitochondrial import inner membrane translocase subunit Tim8	down
Unigene135649	3.2789	0.1166	-4.808641782	4.50777E-08	*Z. mays*	Mitochondrial import receptor subunit TOM40 homolog 1 , TOM40-1	down
Unigene134394	4.6084	0.198	-4.535623287	8.62096E-07	*P. sojae*	Permeases of the drug/metabolite transporter	down
Unigene130658	10.188	0.6179	-4.038123628	7.21353E-23	*P. infestans*	Permeases of the major facilitator superfamily	down
Unigene134000	5.0186	0.2907	-4.104466123	2.45664E-20	*C. subellipsoidea*	Permeases of the major facilitator superfamily	down
Unigene135127	8.4068	0.7149	-3.550573629	4.09337E-20	--	Permeases of the major facilitator superfamily	down
Unigene134817	5.027	0.3898	-3.669479034	4.75914E-17	*E. siliculosus*	Permeases of the major facilitator superfamily	down
Unigene132992	4.7804	0.5	-3.251830321	7.87774E-14	*Z. mays*	Permeases of the major facilitator superfamily	down
Unigene133941	5.212	0.2028	-4.678581241	1.86139E-13	*P. sojae*	Permeases of the major facilitator superfamily	down
Unigene132099	6.0957	0	-6.565370631	8.08715E-13	*C. variabilis*	Permeases of the major facilitator superfamily	down
Unigene134392	5.9599	0.8332	-2.833303836	9.59303E-11	*E. siliculosus*	Permeases of the major facilitator superfamily	down
Unigene133148	4.2717	0.3146	-3.758015709	6.76283E-10	--	Permeases of the major facilitator superfamily	down
Unigene132923	4.7535	0.4201	-3.494981303	4.16201E-08	*P. trichocarpa*	Permeases of the major facilitator superfamily	down

Unigene134838	4.2158	0.2898	-3.857551382	1.42113E-07	*Z. mays*	Permeases of the major facilitator superfamily	down
Unigene132349	3.6831	0	-5.342978209	2.42469E-06	--	Permeases of the major facilitator superfamily	down
Unigene135608	3.8788	0.6059	-2.673126811	2.65239E-06	*Z. mays*	Permeases of the major facilitator superfamily	down
Unigene133317	2.1013	0	-4.857551382	8.13932E-05	*Z. mays*	Permeases of the major facilitator superfamily	down
Unigene130803	6.6903	0.5608	-3.571247197	3.51623E-25	*T. pseudonana*	Nucleoside permease	down
Unigene131614	2.6652	0.2617	-3.342978209	3.02654E-05	*H. vulgare*	solute carrier family 25 (mitochondrial phosphate transporter), member 3	down
Unigene135394	12.0872	0.6165	-4.287964107	1.98385E-62	*S. moellendorffii*	solute carrier family 39 (zinc transporter), member 1/2/3	down
Unigene20264	0.7218	0	-5.120585788	1.37805E-05	*V. vinifera*	Thylakoid ADP,ATP carrier protein, chloroplastic , TAAC	down
Unigene134576	10.1146	0.4438	-4.505249638	2.37545E-22	*H. vulgare*	Importin subunit alpha-1b	down
Unigene134742	11.1876	0.4523	-4.620512185	7.21283E-64	*H. vulgare*	solute carrier family 25 (mitochondrial phosphate transporter), member 3	down
Unigene37931	22.5427	2.2188	-3.339539125	2.01222E-77	*N. scintillans*	solute carrier family 30 (zinc transporter), member 2	down
**ABC transporters**							
Unigene147662	0.001	0.18235	7.510566389	0.006816782	*N. tabacum*	ABC transporter	up
Unigene96594	0.001	0.3222	8.331812684	0.006788476	*P. trichocarpa*	ABC transporter B family member 10, ABCB10	up
Unigene123809	0.0453	0.3635	3.219264215	0.000110988	*C. roseus*	ABC transporter C family member 13, ABCC13	up
Unigene149995	0.1034	4.4084	5.406891218	1.99561E-11	*A. thaliana*	ABC transporter F family member 1, ABCF1	up
Unigene151966	1.2695	7.2043	2.464376713	3.64862E-08	--	ABC transporter	up

Unigene133885	4.8819	0	-5.950660786	5.42407E-09	*C. variabilis*	ABC transporter A family member 1, ABCA1	down
Unigene135275	7.7186	0.5305	-3.857551382	7.18739E-23	*E. siliculosus*	ABC transporter B family member 13, ABCB13	down
Unigene133914	3.2945	0.1477	-4.474222743	1.56486E-06	*P. patens*	ABC transporter B family member 15, ABCB15	down
Unigene139010	9.9272	1.724	-2.535623287	4.93075E-14	*V. vinifera*	ABC transporter B family member 19, ABCB19	down
Unigene132470	3.6422	0.2889	-3.651100505	1.54124E-06	*R. communis*	ABC transporter B family member 24, ABCB24	down
Unigene135276	6.0423	0.1832	-5.038123628	5.23183E-25	*M. pusilla*	ABC transporter B family member 29, ABCB29	down
Unigene131796	4.6669	0.2187	-4.410092405	3.57255E-11	*C. variabilis*	ABC transporter B family member 7, ABCB7	down
Unigene134071	3.0221	0.3462	-3.120585788	4.4579E-06	*A. thaliana*	ABC transporter C family member 12, ABCC12	down
Unigene134070	2.3865	0.164	-3.857551382	0.00019832	*P. sojae*	ABC transporter C family member 5, ABCC5	down
Unigene132149	2.9418	0	-4.758015709	0.000148525	*V. vinifera*	ABC transporter E family member 2, ABCE2	down
Unigene134399	3.8925	0.1982	-4.290510789	4.55794E-19	*V. carteri*	ABC transporter F family member 1, ABCF1	down
Unigene10365	74.5911	4.9959	-3.893301407	0	*H. vulgare*	ABC transporter F family member 4, ABCF4	down
Unigene10876	19.1765	1.5266	-3.600002515	9.4017E-103	*V. vinifera*	ABC transporter G family member 11, ABCG11	down
Unigene113882	2.9164	0.5197	-2.494981303	3.23228E-06	*R. communis*	ABC transporter G family member 34, ABCG34	down
Unigene50467	15.0831	1.132	-3.730729883	1.9222E-166	*R. communis*	ABC-type dipeptide transport system, periplasmic component	down

Unigene132535	3.1014	0.1453	-4.410092405	2.84625E-06	*O. sativa*	ABC-type dipeptide transport system, periplasmic component	down
Unigene129579	33.8268	2.7462	-3.617417378	1.8024E-99	*P. sojae*	ABC-type dipeptide transport system, periplasmic component	down
Unigene133510	15.0168	1.3335	-3.488094917	6.90384E-41	*P. infestans*	ABC-type dipeptide transport system, periplasmic component	down
Unigene136125	2.8706	0	-5.705548289	8.28913E-08	*H. vulgare*	ABC-type multidrug transport system, permease component	down
Unigene130918	7.0554	0.2538	-4.79196304	4.7894E-21	*Micromonas sp.*	ABC-type uncharacterized transport system, permease component	down
Unigene131899	3.8082	0	-6.376925541	1.68796E-11	*P. vulgaris*	ATP-binding cassette, subfamily B (MDR/TAP), member 1	down
Unigene130686	3.5797	0.205	-4.120585788	3.17901E-05	*N. tabacum*	Pleiotropic drug resistance protein 3, PDR3	down
Unigene54592	0.4815	0.0583	-3.120585788	0.000179145	*V. vinifera*	Na+-driven multidrug efflux pump	down
**Amino acid transporters**							
Unigene152266	0.4207	5.0294	3.57290117	1.11762E-10	--	Amino acid transporter	up
Unigene152088	0.4475	5.35	3.57290117	5.09293E-06	*P. patens*	Amino acid transporter	up
Unigene150317	0.1755	3.4666	4.297266727	1.09418E-05	--	Amino acid transporter	up
Unigene152018	0	3.2695	4.856694136	0.000107859	*V. mungo*	Amino acid transporter	up
Unigene152703	0	4.3088	5.371267308	2.84275E-06	*Z. mays*	Ammonia permease	up
Unigene91442	0.378	1.8434	2.322357708	5.00895E-09	*R. communis*	Lysine histidine transporter 1, LHT1	up
Unigene136715	2.0996	0	-5.474222743	7.76128E-07	*T. pseudonana*	Amino acid permeases	down
Unigene133103	2.8461	0	-5.474222743	7.76128E-07	--	Amino acid permeases	down
Unigene134389	10.0232	0.6183	-4.013670584	4.1858E-50	*P. patens*	Amino acid transporters	down
Unigene134560	3.2922	0.5304	-2.628732692	4.59074E-06	*C. variabilis*	Amino-acid permease BAT1 homolog, BAT1	down
Unigene134559	9.5878	0.4707	-4.342978209	1.78478E-33	*B. distachyon*	Amino-acid permease BAT1 homolog, BAT1	down
Unigene19640	1.7934	0.3414	-2.272588881	2.46012E-06	*A. lyrata*	Lysine histidine transporter 1, LHT1	down

Unigene116627	45.948	2.1327	-4.423623989	0	*P. patens*	Cationic amino acid transporter 2, CAT2	down
Unigene102257	21.6736	1.3807	-3.965307562	7.2498E-164	*P. patens*	Cationic amino acid transporter 9, CAT9	down
**Others**							
Unigene4864	0.001	0.1282	7.002252452	0.0141614	*V. vinifera*	Auxin transport	up
Unigene39089	1.4745	6.9057	2.119728541	2.55553E-06	*V. vinifera*	Probable auxin efflux carrier component 6, PIN6	up
Unigene151134	0.3993	3.7355	3.219264215	2.20234E-06	*V. carteri*	Protein ycf2	up
Unigene23038	0.001	0.17965	7.489045125	0.006838707	*V. vinifera*	Probable aquaporin PIP2-5	up
Unigene150611	0.0939	4.784	5.664049058	2.05071E-13	*A. thaliana*	Peptide transporter PTR1	up
Unigene151950	0	3.218	4.856694136	0.000107859	*Zea mays*	other	up
Unigene44342	0.4004	3.7765	3.239163772	5.94545E-15	*P. trichocarpa*	other	up
Unigene135765	3.3344	0	-5.594516976	2.51971E-07	*T. oceanica*	other	down
Unigene133662	4.2026	0.2167	-4.272588881	9.47375E-06	*E. siliculosus*	other	down
Unigene88753	8.1011	1.9841	-2.066138004	1.16748E-05	*P. sitchensis*	other	down
Unigene74909	1537.91	265.7765	-2.429783726	0	*P. trichocarpa*	14 kDa proline-rich protein DC2.15	down
Unigene132878	2.6473	0	-5.808641782	2.76096E-08	*Z. mays*	Ammonium transporter 2 member 1, AMT2-1	down
Unigene86727	25.2727	6.2072	-2.014670026	2.4554E-114	*P. trichocarpa*	Auxin efflux carrier component 2, PIN2	down
Unigene86728	27.9208	4.5941	-2.595648058	8.38867E-92	*P. trichocarpa*	Probable auxin efflux carrier component 2, PIN2	down
Unigene125793	139.926	10.1586	-3.769764667	0	*R. communis*	Heavy metal-associated isoprenylated plant protein 26 , HIPP26	down
Unigene135298	8.7101	0.4869	-4.155775216	9.32163E-37	*C. subellipsoidea*	Metal-nicotianamine transporter YSL2	down
Unigene139560	4.2691	0.8267	-2.32917241	0.000115707	*M. truncatula*	Nucleobase-ascorbate transporter 1 , NAT1	down
Unigene132563	3.7451	0.4065	-3.1985883	9.9151E-05	*R. communis*	Oligopeptide transporter 3, OPT3	down
Unigene134498	3.9202	0	-5.410092405	1.36931E-06	*B. distachyon*	Oligopeptide transporter 7, OPT7	down
Unigene49338	28.0518	2.2785	-3.616470417	4.225E-294	*V. vinifera*	Oligopeptide transporter 7, OPT7	down
Unigene46682	0.1346	0.001	-7.0725346	2.01686E-05	*S. nigrum*	Probable aquaporin SIP2-1	down

Unigene10534	21.0501	1.2813	-4.032891727	2.4676E-229	*G. max*	Probable metal-nicotianamine transporter YSL8	down
Unigene24832	3.7207	0.8256	-2.187699984	2.90809E-05	*P. sojae*	Protein ycf2 (Fragment) ycf2	down
Unigene90644	10.8074	1.9477	-2.466910537	1.43085E-34	*P. sojae*	Putative membrane protein ycf1	down
Unigene136149	3.0916	0	-5.410092405	1.36931E-06	*O. sativa*	other	down

Table S3: Unigenes used in network analysis to generate Fig. 3

Gene	Gene1	Gene2	Score	Annotation
ERF114	Unigene35061_All	Unigene150734_All	0.247453	60S ribosomal protein L27a-3
ERF114	Unigene35061_All	Unigene65397_All	0.247453	60S ribosomal protein L28-1
ERF114	Unigene35061_All	Unigene150479_All	0.247453	Proline-rich receptor-like protein kinase PERK2
ERF114	Unigene35061_All	Unigene74826_All	0.247453	60S ribosomal protein L30
ERF114	Unigene35061_All	Unigene150439_All	0.247453	60S acidic ribosomal protein P1
ERF114	Unigene35061_All	Unigene151582_All	0.258721	Extensin
ERF114	Unigene35061_All	Unigene52220_All	0.258721	60S ribosomal protein L35a-1
ERF114	Unigene35061_All	Unigene150906_All	0.242665	Proline-rich receptor-like protein kinase PERK2
ERF114	Unigene35061_All	Unigene150421_All	0.242665	Pectinesterase 2
ERF114	Unigene35061_All	Unigene152320_All	0.29885	Vegetative cell wall protein gp1
ERF114	Unigene35061_All	Unigene76863_All	0.249152	60S ribosomal protein L34
ERF114	Unigene35061_All	Unigene52552_All	0.247453	40S ribosomal protein S26-3
ERF114	Unigene35061_All	Unigene88258_All	0.258721	60S ribosomal protein L35
ERF114	Unigene35061_All	Unigene76954_All	0.237926	60S ribosomal protein L27
ERF114	Unigene35061_All	Unigene88897_All	0.237926	60S ribosomal protein L19-3
ERF114	Unigene35061_All	Unigene65284_All	0.237926	60S ribosomal protein L7a
ERF114	Unigene35061_All	Unigene25154_All	0.249152	Agmatine coumaroyltransferase-2
ERF114	Unigene35061_All	Unigene88252_All	0.237926	60S ribosomal protein L14
ERF114	Unigene35061_All	Unigene64953_All	0.249152	40S ribosomal protein S29
ERF114	Unigene35061_All	Unigene151170_All	0.235708	60S ribosomal protein L6-1
ERF114	Unigene35061_All	Unigene37024_All	0.237926	60S ribosomal protein L17
ERF114	Unigene35061_All	Unigene152338_All	0.258721	Elongation factor 1-gamma 3
ERF114	Unigene35061_All	Unigene23630_All	0.258721	Pollen-specific leucine-rich repeat extensin-like protein 1
ERF114	Unigene35061_All	Unigene150352_All	0.247453	40S ribosomal protein S7
ERF114	Unigene35061_All	Unigene128845_All	0.247453	40S ribosomal protein S10
ERF114	Unigene35061_All	Unigene126725_All	0.237926	40S ribosomal protein S3a-2
ERF114	Unigene35061_All	Unigene88245_All	0.237926	60S ribosomal protein L26-2
ERF114	Unigene35061_All	Unigene116322_All	0.244947	Betaine aldehyde dehydrogenase

ERF114	Unigene35061_All	Unigene90715_All	0.29885	Anther-specific proline-rich protein APG
ERF114	Unigene35061_All	Unigene37646_All	0.237926	Eukaryotic translation initiation factor 5A-1
ERF114	Unigene35061_All	Unigene150933_All	0.247453	60S ribosomal protein L18a
ERF114	Unigene35061_All	Unigene152410_All	0.258721	40S ribosomal protein S23
ERF114	Unigene35061_All	Unigene78218_All	0.235708	Tubulin beta-8 chain
ERF114	Unigene35061_All	Unigene101984_All	0.237926	60S ribosomal protein L9
ERF114	Unigene35061_All	Unigene51833_All	0.237926	60S ribosomal protein L36-1
ERF114	Unigene35061_All	Unigene103611_All	0.289185	Extensin HRGP
ERF114	Unigene35061_All	Unigene74456_All	0.237926	Eukaryotic initiation factor 4A-3
ERF114	Unigene35061_All	Unigene24440_All	0.237926	40S ribosomal protein S16-1
ERF114	Unigene35061_All	Unigene150601_All	0.253737	Nucleolin 1
ERF114	Unigene35061_All	Unigene10207_All	0.237926	60S ribosomal protein L32-1
ERF114	Unigene35061_All	Unigene149930_All	0.235708	MLO-like protein 14
ERF114	Unigene35061_All	Unigene88349_All	0.237926	Elongation factor 1-alpha
ERF114	Unigene35061_All	Unigene151010_All	0.239721	Pentatricopeptide repeat-containing protein
ERF114	Unigene35061_All	Unigene154422_All	0.235708	Protein kinase APK1A
ERF114	Unigene35061_All	Unigene74438_All	0.249152	40S ribosomal protein S15a-3
ERF114	Unigene35061_All	Unigene10777_All	0.237926	40S ribosomal protein S27-2
ERF114	Unigene35061_All	Unigene125771_All	0.237926	ADP,ATP carrier protein 1 ANT1
ERF114	Unigene35061_All	Unigene5741_All	0.235708	U-box domain-containing protein 10
ERF114	Unigene35061_All	Unigene116294_All	0.237926	60S ribosomal protein L37-2
ERF114	Unigene35061_All	Unigene100954_All	0.237926	40S ribosomal protein S4
ERF114	Unigene35061_All	Unigene115565_All	0.276359	60S ribosomal protein L8
ERF114	Unigene35061_All	Unigene51651_All	0.237926	40S ribosomal protein S11
ERF114	Unigene35061_All	Unigene150763_All	0.258721	60S ribosomal protein L29-1
ERF114	Unigene35061_All	Unigene150106_All	0.235708	29 kDa ribonucleoprotein A
ERF114	Unigene35061_All	Unigene103167_All	0.237926	60S acidic ribosomal protein
ERF114	Unigene35061_All	Unigene115161_All	0.237926	40S ribosomal protein S5
ERF114	Unigene35061_All	Unigene60515_All	0.269785	Pyruvate dehydrogenase E1 pbhB
ERF114	Unigene35061_All	Unigene150235_All	0.235708	Cold shock protein 1 CSP1
ERF114	Unigene35061_All	Unigene50333_All	0.237926	40S ribosomal protein S28
ERF114	Unigene35061_All	Unigene90287_All	0.237926	40S ribosomal protein S18
ERF114	Unigene35061_All	Unigene116826_All	0.237926	60S ribosomal protein L39

ERF114	Unigene35061_All	Unigene127331_All	0.29885	Uncharacterized protein At5g39570
ERF114	Unigene35061_All	Unigene150578_All	0.247453	40S ribosomal protein S3-2
ERF114	Unigene35061_All	Unigene51771_All	0.237926	60S ribosomal protein L18
ERF114	Unigene35061_All	Unigene127657_All	0.249152	60S ribosomal protein L10
ERF114	Unigene35061_All	Unigene21151_All	0.237926	Ubiquitin-fold modifier 1
ERF114	Unigene35061_All	Unigene115288_All	0.249152	40S ribosomal protein S15
ERF114	Unigene35061_All	Unigene51535_All	0.237926	40S ribosomal protein S25
ERF114	Unigene35061_All	Unigene76994_All	0.242665	40S ribosomal protein S21
ERF114	Unigene35061_All	Unigene150851_All	0.258721	60S ribosomal protein L5
ERF114	Unigene35061_All	Unigene24245_All	0.247453	60S ribosomal protein L24
ERF114	Unigene35061_All	Unigene150143_All	0.263467	Cold shock domain-containing protein 3 CSP3
ERF114	Unigene35061_All	Unigene103078_All	0.258721	60S ribosomal protein L15-1
ERF114	Unigene35061_All	Unigene116737_All	0.28302	Vegetative cell wall protein gp1
ERF114	Unigene35061_All	Unigene75910_All	0.247453	UDP-glucose 4-epimerase GEPI48
ERF114	Unigene35061_All	Unigene102997_All	0.237926	40S ribosomal protein S20-2
ERF114	Unigene35061_All	Unigene38087_All	0.247453	60S ribosomal protein L11-2
ERF114	Unigene35061_All	Unigene52325_All	0.285952	60S acidic ribosomal protein P2B
ERF114	Unigene35061_All	Unigene76073_All	0.247453	60S ribosomal protein L23A
ERF114	Unigene35061_All	Unigene22872_All	0.237926	Probable mediator of RNA polymerase II transcription subunit 37e
ERF114	Unigene35061_All	Unigene65269_All	0.247453	60S ribosomal protein L6-2
ERF114	Unigene35061_All	Unigene152403_All	0.237926	60S ribosomal protein L18a-2
ERF114	Unigene35061_All	Unigene88787_All	0.237926	ATP synthase subunit 9, mitochondrial
ERF114	Unigene35061_All	Unigene50163_All	0.237926	40S ribosomal protein S6
ARF 25	Unigene60441_All	Unigene63897_All	0.220727	--
ARF 25	Unigene60441_All	Unigene24572_All	0.364412	GDSL esterase/lipase At1g54790
ARF 25	Unigene60441_All	Unigene124338_All	0.337775	Cytochrome P450 94A1
ARF 25	Unigene60441_All	Unigene53113_All	0.359757	Probable ubiquitin-conjugating enzyme E2 23
ARF 25	Unigene60441_All	Unigene30041_All	0.366275	Chaperone protein dnaJ 49
ARF 25	Unigene60441_All	Unigene19670_All	0.362112	Probable eukaryotic translation initiation factor 5-2
ARF 25	Unigene60441_All	Unigene60472_All	0.344505	Nucleolin 2
ARF 25	Unigene60441_All	Unigene25129_All	0.347944	Trehalose-phosphate phosphatase A TPPA

ARF 25	Unigene60441_All	Unigene54967_All	0.343931	Uncharacterized protein At1g10890
ARF 25	Unigene60441_All	Unigene72260_All	0.337938	E3 ubiquitin-protein ligase RMA1
ARF 25	Unigene60441_All	Unigene29962_All	0.344505	COP9 signalosome complex subunit 4 CSN4
ARF 25	Unigene60441_All	Unigene46383_All	0.34985	Allene oxide synthase, chloroplastic CYP74A
ARF 25	Unigene60441_All	Unigene70703_All	0.365227	Ubiquitin carboxyl-terminal hydrolase 16 UBP16
ARF 25	Unigene60441_All	Unigene94122_All	0.371613	Wall-associated receptor kinase-like 10 WAKL10
ARF 25	Unigene60441_All	Unigene148723_All	0.366275	Heparanase-like protein 3
ARF 25	Unigene60441_All	Unigene160190_All	0.218512	--
ARF 25	Unigene60441_All	Unigene106123_All	0.364492	Protein transport protein Sec24-like At3g07100
ARF 25	Unigene60441_All	Unigene47511_All	0.222566	--
ARF 25	Unigene60441_All	Unigene111787_All	0.232815	--
ARF 25	Unigene60441_All	Unigene104096_All	0.364492	Light-regulated protein LIR1
ARF 25	Unigene60441_All	Unigene122545_All	0.326546	4-hydroxybenzoate polyprenyltransferase, mitochondrial
ARF 25	Unigene60441_All	Unigene149724_All	0.377336	Probable peptide/nitrate transporter At3g53960
ARF 25	Unigene60441_All	Unigene64652_All	0.22455	Lignin-forming anionic peroxidase
ARF 25	Unigene60441_All	Unigene47957_All	0.343653	5'-methylthioadenosine/S-adenosylhomocysteine nucleosidase
ARF 25	Unigene60441_All	Unigene48187_All	0.352714	Wall-associated receptor kinase-like 1 WAKL1
ARF 25	Unigene60441_All	Unigene102983_All	0.343653	Probable fructose-bisphosphate aldolase 1, chloroplastic FBA1
ARF 25	Unigene60441_All	Unigene111496_All	0.333764	GDP-L-galactose phosphorylase 1 VTC2
ARF 25	Unigene60441_All	Unigene69228_All	0.343022	DEAD-box ATP-dependent RNA helicase 3, chloroplastic
ARF 25	Unigene60441_All	Unigene120810_All	0.356746	Transcription factor bHLH130
ARF 25	Unigene60441_All	Unigene69859_All	0.223082	--
ARF 25	Unigene60441_All	Unigene76581_All	0.221465	--
ARF 25	Unigene60441_All	Unigene11706_All	0.352046	Alcohol dehydrogenase 3 ADH3
ARF 25	Unigene60441_All	Unigene58511_All	0.359757	Magnesium transporter MRS2-1
ARF 25	Unigene60441_All	Unigene18392_All	0.321764	Soluble inorganic pyrophosphatase IPP
ARF 25	Unigene60441_All	Unigene107038_All	0.333036	Potassium channel SKOR
ARF 25	Unigene60441_All	Unigene39089_All	0.327437	Probable auxin efflux carrier component 6 PIN6
ARF 25	Unigene60441_All	Unigene91364_All	0.343224	Pyruvate dehydrogenase E1 component subunit alpha
ARF 25	Unigene60441_All	Unigene4963_All	0.365227	Isoflavone reductase homolog
ARF 25	Unigene60441_All	Unigene127745_All	0.336485	Beta-D-xylosidase 1 BXL1
ARF 25	Unigene60441_All	Unigene74595_All	0.320924	Cationic peroxidase 1 PNC1
ARF 25	Unigene60441_All	Unigene38541_All	0.340126	Inositol monophosphatase 3 IMP3

ARF 25	Unigene60441_All	Unigene60200_All	0.337775	Probable trans-2-enoyl-CoA reductase, mitochondrial
ARF 25	Unigene60441_All	Unigene8113_All	0.358253	Proline-rich receptor-like protein kinase PERK2
ARF 25	Unigene60441_All	Unigene23891_All	0.229854	Gibberellin 2-beta-dioxygenase 8 GA2OX7
ARF 25	Unigene60441_All	Unigene66618_All	0.361443	Acyl carrier protein 1 ACP1
ARF 25	Unigene60441_All	Unigene60446_All	0.348685	Carbon catabolite repressor protein 4 homolog 4 CCR4-4
ARF 25	Unigene60441_All	Unigene52895_All	0.323784	Ubiquitin-conjugating enzyme E2-23 kDa UBC4
ARF 25	Unigene60441_All	Unigene159085_All	0.314926	Structural maintenance of chromosomes protein 2-2 SMC2-2
ARF 25	Unigene60441_All	Unigene148811_All	0.341788	ENHANCER OF AG-4 protein 2 HUA2
ARF 25	Unigene60441_All	Unigene128334_All	0.341167	Pentatricopeptide repeat-containing protein At4g38010 PCMP-
ARF 25	Unigene60441_All	Unigene102882_All	0.219004	Probable carboxylesterase 9 CXE9
ARF 25	Unigene60441_All	Unigene28590_All	0.354248	Katanin p80 WD40 repeat-containing subunit B1 homolog 1
ARF 25	Unigene60441_All	Unigene24126_All	0.352046	L-ascorbate peroxidase 1, cytosolic APX1
ARF 25	Unigene60441_All	Unigene17647_All	0.340914	MYST-like histone acetyltransferase 2 HAG5
ARF 25	Unigene60441_All	Unigene79681_All	0.339069	Heat shock 70 kDa protein 15 HSP70
ARF 25	Unigene60441_All	Unigene116865_All	0.337775	--
ARF 25	Unigene60441_All	Unigene152117_All	0.358256	Hexokinase-1 HXK1
ARF 25	Unigene60441_All	Unigene80389_All	0.33891	Ubiquitin-conjugating enzyme E2 29 UBC29
ARF 25	Unigene60441_All	Unigene112803_All	0.370016	50S ribosomal protein L12, RPL12-2
ARF 25	Unigene60441_All	Unigene116998_All	0.337648	Putative SWI/SNF-related matrix-associated actin-dependent regulator of chromatin subfamily A member 3-like 3
ARF 25	Unigene60441_All	Unigene43887_All	0.375652	Coatomer subunit gamma-2
ARF 25	Unigene60441_All	Unigene64519_All	0.319665	Probable galacturonosyltransferase 4 GAUT4
ARF 25	Unigene60441_All	Unigene44670_All	0.365227	Mechanosensitive ion channel protein 2, MSL2
ARF 25	Unigene60441_All	Unigene34867_All	0.354248	Bifunctional aspartokinase/homoserine dehydrogenase
ARF 25	Unigene60441_All	Unigene107965_All	0.338339	Dynamin-related protein 3A DRP3A
ARF 25	Unigene60441_All	Unigene25568_All	0.355866	Probable inactive receptor-like protein kinase At3g56050
ARF 25	Unigene60441_All	Unigene110579_All	0.340081	Pyruvate kinase isozyme G, chloroplastic (Fragment)
ARF 25	Unigene60441_All	Unigene112391_All	0.351599	60S ribosomal protein L18a-1
ARF 25	Unigene60441_All	Unigene94521_All	0.228088	--
ARF 25	Unigene60441_All	Unigene66078_All	0.355001	Probable serine/threonine-protein kinase At1g01540
ARF 25	Unigene60441_All	Unigene159439_All	0.31799	5-methyltetrahydropteroyltriglutamate--homocysteine methyltransferase

ARF 25	Unigene60441_All	Unigene57651_All	0.365227	Probably inactive leucine-rich repeat receptor-like protein kinase At5g48380
ARF 25	Unigene60441_All	Unigene147396_All	0.340126	AP-1 complex subunit gamma-2
ARF 25	Unigene60441_All	Unigene94571_All	0.32915	Serine carboxypeptidase-like 13 SCPL13
ARF 25	Unigene60441_All	Unigene52093_All	0.358764	Probable pectinesterase 68 PME68
ARF 25	Unigene60441_All	Unigene56225_All	0.358217	Pto-interacting protein 1PTI1
ARF 25	Unigene60441_All	Unigene97416_All	0.334707	Probable phytol kinase 1, chloroplastic
ARF 25	Unigene60441_All	Unigene101487_All	0.352046	Cytokinin dehydrogenase 1 CKX1
ARF 25	Unigene60441_All	Unigene117440_All	0.339867	Mitogen-activated protein kinase homolog MMK1
ARF 25	Unigene60441_All	Unigene19731_All	0.338317	Cytochrome c
ARF 25	Unigene60441_All	Unigene84237_All	0.34725	Cysteine proteinase RD21A
ARF 25	Unigene60441_All	Unigene98898_All	0.360882	Mitogen-activated protein kinase 8 MPK8
ARF 25	Unigene60441_All	Unigene64991_All	0.335126	Putative SWI/SNF-related matrix-associated actin-dependent regulator of chromatin subfamily A member 3-like 3

Supplemental Table S4

Primer sequences used for qRT-PCR analysis: The unigene sequences obtained by transcriptome analysis were used to design primers with the help of http://www.ncbi.nlm.nih.gov/tools/primer-blast/ online tool and were used in qRT-PCR analysis.

**Unigene name**	**Primer sequence 5'-3'**
Unigene84659<br>*IAA11*	Forward primer GGAGGACAACGACCCGATAC<br>Reverse primer GGCTCATACGCTGGAAGACA
Unigene146802<br>*ABP19a*	Forward primer CGTTATCTGCAGCCCAGCAT<br>Reverse primer TACAAGACGCTTCGTAAGGGG
Unigene152475<br>*GH3.1*	Forward primer AGAATTATCTGCCACCCGGC<br>Reverse primer TGCAGAAGGCCGTCAAAGAT
Unigene16981<br>*AUX22D*	Forward primer AGCCAATTCATCCGGAACCA<br>Reverse primer TCCCAGATGGGGAAGACGAT
Unigene9234<br>*5NG4*	Forward primer GCTGTGGGATTGGAGCACTA<br>Reverse primer ATGCTGGTTCTTGGTGCAGT
Unigene152612<br>*SAM2*	Forward primer ATGGGAGGGAACCGTCGTTA<br>Reverse primer CCAGGGTATCATGTTCGGCT
Unigene150391<br>*ACCox*	Forward primer GGAGGATTTGCCCGTTGTCT<br>Reverse primer GGGCACGGGGGATAGTAATG
Unigene150671<br>*SHMT*	Forward primer TGCGAGCGTAGTCGATTTCA<br>Reverse primer CTGATGGGACTGGATCTGCC
Unigene151662<br>*MethSynI*	Forward primer GACCTGGTTGGAGCCAATCTT<br>Reverse primer AACCTTCCCAAGCTTGTCCTT
Unigene150679<br>*MethSynII*	Forward primer GGTTGGGTCCAGTCTTACGG<br>Reverse primer ACCCTTCATGGGCTTCTTGG
Unigene151933<br>*MAPK3*	Forward primer TCGAAGTGTGTTTTCGCTGC<br>Reverse primer AAATGCTTGAAGGCAAGCCG
Unigene151582<br>*Extensin*	Forward primer CCTTGTTGGAGTGCTCGGTA<br>Reverse primer CAAGGTCTCAACAAAGCCGC

Unigene100648<br>*WRKY9*	Forward primer GCAACAACTTGACGGGCAAT<br>Reverse primer CGGTGCAACGATAGTAGGCT
Unigene8314<br>*WRKY22*	Forward primer CATGTACCCGCCGAGAAGTT<br>Reverse primer CGCTCCACTTGTTTTCTCGC
Unigene35061<br>*ERF114*	Forward primer AGCTCAACTTCCCCGAAAGG<br>Reverse primer GCATGAGGTCTGCGTAGTGA
Unigene 42955<br>*ERF1B*	Forward primer GACTAAGGCCAGGCAGTACA<br>Reverse primer CTCTTCCACGCCACACTTCA
Unigene119295<br>*ARF1*	Forward primer ATCTGCATGGAACCGAGTGG<br>Reverse primer GGCATGGAGCTTTGTTGACG
Unigene26596<br>*ARF6*	Forward primer TGCATTTATGCGTTTCTCGCC<br>Reverse primer GATTTTGGTGACGGACTGCG
Unigene60441<br>*ARF25*	Forward primer GAGTACTGGTGTCGCTTGCT<br>Reverse primer CCCAAGCTTAACGCCACAAC
Unigene152309<br>*NAC2a*	Forward primer GCATTGAAGAGCTCCCCGAC<br>Reverse primer CTTGCGAGCCTTCTTCTCGT
Unigene42722<br>*NAC2b*	Forward primer GCTGTTGCTCTTTTTCCGGG<br>Reverse primer CACCCAAAGCCTGTCGGTAT
Unigene113286<br>*Trihelix GT-3b*	Forward primer GTTGGATGAATTTCCGCCGC<br>Reverse primer ACGAGAACGAAGACAGCGAG
Unigene120810<br>*bHLH130*	Forward primer CTGGGAGCAGTCCTCCTTTG<br>Reverse primer CAACACCACGAGTGACGGTA
Unigene20461<br>*bHLH137*	Forward primer TATCCTCCAACGGTGATGGC<br>Reverse primer ACTGGAAAGGCCCTTGTGTT
Unigene137386<br>*WRKY50*	Forward primer GGACGACGGATATAAGTGGAGG<br>Reverse primer GCTAGGATCCCCCTTTTCCC
Unigene131052<br>*Zinc finger11*	Forward primer TAGACGCCGGACGTTTATCG<br>Reverse primer AAACCTTCCGTACCCAACCC
Unigene121384<br>*MYB86*	Forward primer ATTCAGGTGCTGGAAGGACG<br>Reverse primer GCCCAGAACTCGAACCAAGA
Unigene10919	Forward primer CAATGTCGTTCGCCCGAAAA

GATA4	Reverse primer TTTTGTGTACCCGCTGACGA
Unigene29962<br>*CSN4*	Forward primer TGGGCCTTCGTAGCTACTCT<br>Reverse primer GCGGTATTAGACCTCCGAGC
Unigene39089<br>*PIN6*	Forward primer AGGGGTTGTTCTGGGAGATGA<br>Reverse primer CAAAGTGATGTGCGCAATGGT
Unigene107038<br>*SKOR*	Forward primer ACCGTCATGCTTAGCCTTTGA<br>Reverse primer CGGACCCGAGTTACCTACAGA
Unigene116322<br>*BADH*	Forward primer CACCCTACCATCTACCGCAC<br>Reverse primer AGAAGTTACCAGCCGCATCC
Unigene150235<br>*CSP1*	Forward primer AGTGCTATAACTGCGGCGAG<br>Reverse primer GGCGCAATCTCACACAATCG
Unigene152338<br>*EF1*	Forward primer CTTCCTGCTTTCGATGTCGC<br>Reverse primer TTGAACACCTTACCGTCGGC
Unigene60515<br>*PDH*	Forward primer GTGACCAAGGGCTCGTCTAC<br>Reverse primer GCATGCCACAGTTGTTGGAG
AtPIN6	Forward primer AGTCCTAGAAGGCTCTCGGG<br>Reverse primer TTTGCCCAGACTCCCACATC
AtSKOR	Forward primer TCTCTTCGTTTCCGAGTGGC<br>Reverse primer TTCAGCTGCTCCTTTGCTGT
AtMPK8	Forward primer ACCAGCTTCTTCGTGGTCTG<br>Reverse primer ATATAGCGGTTGGGGCATCG
AtCSDP	Forward primer TTCAGAGGATCAATCGGCGG<br>Reverse primer TTCAGAGGATCAATCGGCGG
AtPDH	Forward primer AGGTACCATGTGATGCAGGC<br>Reverse primer CAGCTGTAAGACTTGCCCCA
AtERF115	Forward primer GTGGCTCGGGACATTTGAGA<br>Reverse primer TTGCGGATTAGTCTGCGGAT
AtRD29A	Forward primer GGATCAAACAGAGGAACCACC<br>Reverse primer CCTCCGATGCTGGAACATTAGT
AtNAC2	Forward primer AATGGCGGCTGCTTCTACAG<br>Reverse primer TCGCCATCATCATAGGACCTG

AtHAK5	Forward primer TTATGTCCCTTCATCGGGGC<br>Reverse primer TTCTCTGCCGTAATCTCGGTC
AtHKT1	Forward primer AGTAGACCTCTCTACACTTTCC<br>Reverse primer TTGACCATCATACTCATTGCTG
Unigene149995	Forward primer AAGCGCAGTTCGAGAATCCA<br>Reverse primer ACCTTCCGTTTCGCTGATGT
Unigene115940	Forward primer AAGAAGTTCAAGCAGCGGGT<br>Reverse primer TGTCTGCAATACCGGACACC
Unigene104256	Forward primer TTTGAGGTGGGCTGTTGTCC<br>Reverse primer TTTGCTTTTGCTCACCGCAA
Unigene124338	Forward primer CGCACTCTCGAGAATCGGAA<br>Reverse primer CTTTCTGGGAACGGGCATCT
Unigene28507	Forward primer TCTGTGCTATCCCACACCCT<br>Reverse primer AAGAGGATGCTCACCCAAGC
Unigene57507	Forward primer ACACAGTGGAAGCATCAGGG<br>Reverse primer TTTGCTGGGATGGTGCTAGG
Unigene74595	Forward primer CGAGAGGTTGTTGTTTCCGC<br>Reverse primer GAGGTGCGTGACTTTCAGGA
Unigene87779	Forward primer GAGGGAAGTGTCCCGTTGAG<br>Reverse primer GACTAATCTCTCCGGCGCTC
Unigene77474	Forward primer ATCGAGCTGAACACGAACGA<br>Reverse primer GTCAGCACGTCCTGCAATTC
Unigene150442	Forward primer CATTGCCCGTATTGCCTTCG<br>Reverse primer GCCATACTTCTCAACCGCCT
Unigene74868	Forward primer GCCAAGTTAACCTGCTCCCT<br>Reverse primer CAGTGAAGGGAAGCTTCGGA
Unigene53147	Forward primer GGCGGTAACCACTTTCTCCA<br>Reverse primer ATCAGTCTCACATCCGCAGC
Unigene152338	Forward primer CTTCCTGCTTTCGATGTCGC<br>Reverse primer TTGAACACCTTACCGTCGGC

Unigene150420	Forward primer TGTGGGGATGGTTTGTAGCG<br>Reverse primer AACAGCTTTGACTCCTGGGG
Unigene151507	Forward primer CGCCGACGTTGAAGCTAAAG<br>Reverse primer GCCGTGACACGAGATAAGGT
Unigene150479	Forward primer CTCAGCTTTTGTCCGTCCCT<br>Reverse primer CCGATGAACGAGACGACCAT
Unigene10365	Forward primer CTTGACACCCTCGACAACGA<br>Reverse primer CACCACCTCGACAAGACTCC
Unigene126523	Forward primer TTTTTGTGTACAGGGCCCCA<br>Reverse primer TGCTACAGCTACCCCTTTTGG
Unigene87838	Forward primer ACGATCCTGAGCAGAGACCT<br>Reverse primer TGACAACCGTCTGTTGGGAG
Unigene129669	Forward primer AAACCCTTCCATCGGAACCC<br>Reverse primer TGACAAACTCGTCGGTGAGG
Unigene133874	Forward primer ATTGCTATCCCAGCGAGTGG<br>Reverse primer ACCATTCGTTCCCTAGCAGC
Unigene22932	Forward primer CGGCCTTTCTCGTCCTGAAT<br>Reverse primer CACCCAATCCGTTTCCCTCA
Unigene6501	Forward primer GTCACCAGCTGAAGAGGGTC<br>Reverse primer CTTGCCGTTTTGGAACTGGG
Unigene9466	Forward primer TGATCTTAGGCTGGCTCCCT<br>Reverse primer CCGACAAGTCCACTCCACAT
Unigene134549	Forward primer TGATGTCGGTGTGTCTCGTG<br>Reverse primer CGAGCTCATCTCGTCAGCAT
Unigene134562	Forward primer AGCTAGACGTCGAACCGTTG<br>Reverse primer GTATGCGCAAGCTCATCGTC
Unigene134283	Forward primer TGAGTTCCGTAGATTGGCCG<br>Reverse primer TTCCGTGCTACCGGAAACTG
Unigene134246	Forward primer AGGTACGAGGTGCGTAGACT<br>Reverse primer CAGGCCATGACTGACCTTGT
Unigene134456	Forward primer GCCGAGCTCGAGAAGTTGTA

	Reverse primer ACCTGGTCCATAGTTGCGTC
Unigene166335	Forward primer AGGTCAGCTGAATCTCGGTG<br>Reverse primer TCCCAATCCGACATTTTGGCA
Unigene125764	Forward primer GTGCTCGACCCCATCTTCAA<br>Reverse primer TAACCTTGAGGAGCGCCTTG
Unigene49875	Forward primer CCAGTTCATCACCGCCGATA<br>Reverse primer GTTGGTGTCGCTGAATGCTG
Unigene75887	Forward primer AGTTGAGAAGACCGCGGAAG<br>Reverse primer ACACGTACACGACCAACCTC
Unigene89776	Forward primer TACAGAGATTTGGCCCTGCG<br>Reverse primer TCTGCATCTGCCTACCTCCT
Unigene69183	Forward primer AGGACGAGGGTGCAAAATCC<br>Reverse primer GTGAGCGGGTAACAAGACGA
Unigene93351	Forward primer GTGCCTCTGAACCGACTGAA<br>Reverse primer AGAGCCAAGAACTTGGACGG
Unigene64865	Forward primer CTCAGGAAGAAGGATGGGTCG<br>Reverse primer CAATGGAGGTTGGAGAAGGCT
Unigene92785	Forward primer CGGCACATACAGACCCCAAT<br>Reverse primer AAGCATCTGCCTGGGGTTTT
Unigene164657	Forward primer GGCCAACGGGTGATAAGAGT<br>Reverse primer ACATCGCTCCTGGCAGTTAG
Unigene31992	Forward primer CCAAGCACTGACCCATCCAT<br>Reverse primer AAGCGCTTTTAAGGCAGCAC
Unigene74595<br>*Cationic peroxidase*	Forward primer GAGTCCACGGCTGTCTTGAT<br>Reverse primer TAAGACCATGGCCACCACTG
Unigene*42955*<br>*ERF1B*	Forward primer GACTAAGGCCAGGCAGTACA<br>Reverse primer CTCTTCCACGCCACACTTCA
Unigene35061<br>*ERF114*	Forward primer CGCATTCTCGCTTGCTACAC<br>Reverse primer GGATCCACCGTGTCTGACTG
Unigene152117<br>*Hexokinase*	Forward primer CCAAGCTCTTCATCTGCCGT<br>Reverse primer CCCACCAAGAAAAGCAAACCA

Unigene113286<br>*Trihelix*	Forward primer CCCCGTCCTCAATCCTCTCT<br>Reverse primer GACCTGCTGTCTGGTCTTGT
Unigene150075<br>*14-3-3*	Forward primer AGTTCAGTGCAAGACCGAGG<br>Reverse primer GTTGGTGACAAGCGCAAGAC
Unigene3470<br>*VHA-C*	Forward primer GCGATATGGTCTGCCTCCAT<br>Reverse primer AGCGACACTACACTGCCAAA
Unigene29379<br>*VPP*	Forward primer GAGCTTGACCTTGGACACGA<br>Reverse primer CCCGACTTCACGGCTATCTG
Unigene69535<br>*CBL*	Forward primer ATGTCTCCACTTGGCTCACG<br>Reverse primer TTCTTCCGCCAATCGGTCAA
Unigene51908<br>*NHX2*	Forward primer AACGGTCTCTTCAGACGCAAT<br>Reverse primer TGTCTACCTTCACGAGGTTCG
Unigene115806<br>*NHX6*	Forward primer GACGCCCTACTTGCACAAATC<br>Reverse primer TGCTGCATCGTTTACTGAATTTGA
Unigene123012<br>*CIPK*	Forward primer TGGCAGAAGCTACCAAGTGT<br>Reverse primer CCTCGGGACGGAATCTTCTT
Unigene151915<br>*Calmodulin*	Forward primer GCTTTTCTGTCCTGACCGCC<br>Reverse primer AGGCCTCCTTAAACTCGGAGA
